# SIPSim: A Modeling Toolkit to Predict Accuracy and Aid Design of DNA-SIP Experiments

**DOI:** 10.3389/fmicb.2018.00570

**Published:** 2018-03-28

**Authors:** Nicholas D. Youngblut, Samuel E. Barnett, Daniel H. Buckley

**Affiliations:** School of Integrative Plant Science, Cornell University, Ithaca, NY, United States

**Keywords:** DNA-SIP, SIP, method, microbial, community, function, SIPSim

## Abstract

DNA Stable isotope probing (DNA-SIP) is a powerful method that links identity to function within microbial communities. The combination of DNA-SIP with multiplexed high throughput DNA sequencing enables simultaneous mapping of *in situ* assimilation dynamics for thousands of microbial taxonomic units. Hence, high throughput sequencing enabled SIP has enormous potential to reveal patterns of carbon and nitrogen exchange within microbial food webs. There are several different methods for analyzing DNA-SIP data and despite the power of SIP experiments, it remains difficult to comprehensively evaluate method accuracy across a wide range of experimental parameters. We have developed a toolset (SIPSim) that simulates DNA-SIP data, and we use this toolset to systematically evaluate different methods for analyzing DNA-SIP data. Specifically, we employ SIPSim to evaluate the effects that key experimental parameters (e.g., level of isotopic enrichment, number of labeled taxa, relative abundance of labeled taxa, community richness, community evenness, and beta-diversity) have on the specificity, sensitivity, and balanced accuracy (defined as the product of specificity and sensitivity) of DNA-SIP analyses. Furthermore, SIPSim can predict analytical accuracy and power as a function of experimental design and community characteristics, and thus should be of great use in the design and interpretation of DNA-SIP experiments.

## Introduction

Stable isotope probing of nucleic acids (DNA-SIP and RNA-SIP) is a powerful culture-independent method for linking microbial metabolic functioning to taxonomic identity (Radajewski et al., 2003). In particular, DNA-SIP has been used in a multitude of environments to identify microbial assimilation of various ^13^C- and ^15^N-labeled substrates into DNA (Uhlík et al., 2009). DNA-SIP identifies microbes that assimilate these isotopes into their DNA (“incorporators”) by exploiting the increased buoyant density (BD) of isotopically labeled (“heavy”) DNA relative to unlabeled (“light”) DNA. For example, fully ^13^C- and ^15^N-labeled DNA will increase in BD by 0.036 and 0.016 g ml^−1^, respectively (Birnie and Rickwood, 1978).

Ideally, isopycnic centrifugation could be used to completely separate labeled and unlabeled DNA fragments based solely on this difference in BD. However, several factors besides BD can influence the position of DNA in isopycnic gradients. For example, G + C content variation within a single genome can produce unlabeled DNA fragments that vary in BD by up to 0.03 g ml^−1^, while G + C content variation between microbial genomes can cause the average BD of unlabeled DNA fragments to vary by up to 0.05 g ml^−1^ (Youngblut and Buckley, 2014). In addition, DNA in SIP experiments will often be partially labeled as a consequence of isotope dilution from unlabeled endogenous substrates. Therefore, it is unlikely that nucleic acid SIP experiments will ever achieve complete separation of labeled and unlabeled DNA.

In the absence of complete separation between labeled and unlabeled DNA, isotope incorporators must be identified using some statistical procedure suitable for comparing the BD distributions of DNA fragments from labeled treatment samples and unlabeled control samples (Pepe-Ranney et al., 2016a). The use of multiplexed high throughput sequencing with DNA-SIP makes it possible to sequence SSU rRNA amplicons across many density gradient fractions and simultaneously determine the BD distributions for thousands of taxa. The problem then becomes one of identifying those taxa that have increased in BD in the isotopically labeled samples relative to the corresponding unlabeled controls.

Different analytical approaches have been applied to DNA-SIP datasets to identify DNA sequences of ^13^C-labeled taxa. The earliest and simplest approach to identifying ^13^C-labeled DNA sequences (described herein as Heavy-SIP) is to identify SSU rRNA amplicons that occur in “heavy” fractions of CsCl gradients containing ^13^C-labeled DNA, but do not occur in either “light” fractions or in “heavy” fractions of unlabeled control gradients (Radajewski et al., 2003; Lueders et al., 2004). More recent approaches include “high resolution stable isotope probing” (HR-SIP) and “quantitative stable isotope probing” (qSIP), which both analyze SSU rRNA amplicons across numerous gradient fractions (Hungate et al., 2015; Pepe-Ranney et al., 2016a,b). All of these methods differ in the statistical procedures used to detect taxa that incorporate isotopic label. Heavy-SIP often employs either *t*-test, Fisher's exact test, or analogous approaches to compare OTU relative abundance between pairs of fractions (e.g., heavy vs. light). HR-SIP identifies isotopically labeled taxa by evaluating the sequence composition of several high density “heavy” fractions using a differential abundance quantification framework that evaluates sequence count data in isotopically labeled samples relative to their corresponding unlabeled controls. Differential abundance between the “heavy” fractions of labeled and control gradients is tested with DESeq2 (Love et al., 2014), which uses sophisticated statistical methods to reduce technical error and increase analytical power for analysis of microbiome data (McMurdie and Holmes, 2014). In qSIP, SSU rRNA relative abundance values are transformed using qPCR estimates of total SSU rRNA gene copies present within gradient fractions. These normalized data are used to determine the weighted average BD for each taxon in both isotopically labeled samples and corresponding unlabeled controls (Hungate et al., 2015). Incorporators are then determined by using a permutation procedure using 90% confidence intervals to identify those taxa whose BD shifts are unlikely to occur as a result of chance.

While DNA-SIP is a powerful method for the discovery and characterization of microorganisms *in situ*, systematic assessment of the specificity or sensitivity of this method has not been performed. Empirical validations of DNA-SIP methods typically include only one or a few organisms or simple mock communities (Lueders et al., 2004; Buckley et al., 2007; Cupples et al., 2007; Wawrik et al., 2009; Andeer et al., 2012), and such approaches do not adequately replicate the complexity of the DNA fragment BD distributions expected in a typical DNA-SIP experiment (Youngblut and Buckley, 2014). DNA-SIP experiments vary in the diversity of the target community, DNA G + C content distribution, the number of incorporators, incorporator relative abundance, and the atom % excess of labeled DNA. Systematic evaluation of method accuracy should address the effects that all of these variables have on the sensitivity and specificity of detecting isotope incorporators. Since DNA-SIP experiments are costly, technically difficult, and laborious, it is not practical to perform empirical assessment across this full range of variables.

Fortunately, the physics of isopycnic centrifugation have been well characterized mathematically, and the behavior of individual DNA fragments in CsCl gradients is highly reproducible and predictable from first principles (Meselson et al., 1957; Fritsch, 1975; Birnie and Rickwood, 1978). In addition, genome sequences are available for thousands of diverse microorganisms, and these genomes can be used to simulate DNA fragments representative of community DNA (Youngblut and Buckley, 2014). Hence, we can simulate realistic DNA-SIP data for *in silico* microbial communities that differ in diversity (richness, evenness, and composition), where the relative abundance, genome G + C content, and atom % isotope enrichment are defined for discrete DNA fragments from every genome. We have developed a computational toolset for simulating DNA-SIP data (SIPSim) and used this simulation framework to systematically and objectively evaluate how changes in key SIP experimental parameters are predicted to affect DNA-SIP accuracy.

## Methods

### Theory underlying the simulation framework

DNA stable isotope probing employs isopycnic centrifugation to separate isotopically enriched (“heavy”) DNA molecules from unlabeled (“light”) DNA based on their differences in buoyant density (BD). Isopycnic centrifugation is distinguished from other centrifugation methods in that centrifugation is carried out long enough to both generate a density gradient (typically using CsCl for DNA-SIP) and allow all macromolecules of interest reach sedimentation equilibrium, which is the point at which sedimentation rates equal rates of diffusion (Hearst and Schmid, 1973; Birnie and Rickwood, 1978). Empirical studies have shown that the average BD (ρ) of a mixture of DNA molecules is linearly related to the average G + C content for that collection of molecules:

(1) ρ= 0.098 [G+C] + 1.66

where [G + C] is the mole fraction of genome G + C content (Schildkraut et al., 1962; Birnie and Rickwood, 1978). In addition, empirical studies have also shown that homogeneous mixtures of DNA molecules form a Gaussian distribution in an isopycnic gradient when at sedimentation equilibrium (Meselson et al., 1957; Fritsch, 1975). Therefore, in order to model the BD distribution of a heterogeneous set of genomic DNA fragments, a Gaussian distribution must be estimated for each homogeneous subset of molecules rather than using discrete BD values (as described in Supplementary Material). Based on the work of Meselson et al. (1957), Fritsch (1975) derived an equation describing time to reach sedimentation equilibrium, which can be reworked to calculate the standard deviation (σ) of the Gaussian distribution:

(2) $$\sigma =\;\frac{L}{e^{\left(\gamma -\;1.26\right)}}$$

(2.1) $$\gamma =\frac{t\omega^{4}r_{p}^{2}s}{\beta (p_{p}-p_{m})}$$

where *L* is the effective length of the gradient (cm), *t* is time in seconds, ω is the angular velocity (radians sec^−1^), *r*_*p*_ is the distance of the particle from the axis of rotation (cm), *s* is the sedimentation coefficient of the particle, β° is the coefficient specific to the density gradient medium (e.g., CsCl); *p*_*p*_ and *p*_*m*_ are the maximum and minimum distances between the gradient and axis of rotation (cm) (Fritsch, 1975). By assuming that sedimentation equilibrium has been reached for all macromolecules of interest, Clay and colleagues derived a simplified equation for determining σ from the calculations in Schmid and Hearst (1972):

(3) $$\sigma =\sqrt{\frac{\rho RT}{\beta^{2}GM_{C}l}}$$

where ρ is the BD of the particle, *R* is the universal gas constant, *T* is the temperature in Kelvins, β is a proportionality constant for aqueous salts of specific densities, *G* is a buoyancy factor as described in Clay et al. (2003), *M*_*C*_ is the molecular weight per base pair of DNA, and *l* is the fragment length (bp). For most DNA-SIP experiments, the assumption of sedimentation equilibrium for all DNA fragments is likely to be unrealistic for relatively short DNA fragments (e.g., <4 kb), given that the time to reach equilibrium is inversely proportional to diffusion and hence rises dramatically with decreasing fragment length (Meselson et al., 1957; Birnie and Rickwood, 1978; Youngblut and Buckley, 2014). However, the ultracentrifugation durations used in typical DNA-SIP experiments should still generally produce small σ values for short DNA fragments according to Equation (2) (Neufeld et al., 2007). Therefore, Equation (3) provides a good approximation for modeling the BD distribution of DNA in density gradients generated in typical DNA-SIP experiments.

The distribution of a heterogeneous mixture of DNA fragments in an isopycnic gradient can thus be modeled by integrating the Gaussian distributions of each homogeneous subset of DNA fragments, where the mean of each Gaussian is determined by Equation (1) and the standard deviation derived from Equation (3). In this way, the BD distribution for a given genome in an isopycnic gradient can be modeled by the following steps: simulate genome fragmentation resulting from DNA extraction, bin gDNA fragments with respect to length and G + C content, model Gaussian distribution for each fragment bin, and then integrate these distributions to describe the cumulative DNA distribution in fractions of the gradient. A strictly Gaussian model predicts that, within a CsCl density gradient, DNA fragments should be undetectable (i.e., probability density < 10^−7^) in factions of both high and low BD (Figure S1). However, empirical observations show DNA to occur throughout CsCl gradients (Birnie and Rickwood, 1978; Lueders et al., 2004; Leigh et al., 2007). We were able to reconcile the difference between observed and expected DNA distributions as a function of fluid mechanics during gradient reorientation (as described below and in Supplementary Material).

Gradient reorientation impacts the BD distribution of DNA. During isopycnic centrifugation, the buoyant density gradient forms perpendicular to the axis of rotation (Figure S2), and gradient reorientation during centrifuge deceleration is dramatic, especially for vertical rotors (Flamm et al., 1966). While the distortion of the BD gradient during reorientation has been shown to be minimal in the aggregate (Fisher et al., 1964; Flamm et al., 1966), the inevitable presence of a diffusive boundary layer along the tube wall is sufficient to entrain quantities of DNA, which are small but should be readily detectable by high throughput sequencing methods. The flow field that occurs during gradient reorientation entrains along the tube wall a volume with a dimension proportional to flow velocity, fluid viscosity, and surface topography (Tritton, 1977; Cohen and Dowling, 2012). Following gradient reorientation, DNA from the entrained volume will combine with DNA from the reoriented volume, thereby introducing a small amount of non-BD-equilibrium DNA into each gradient fraction (Figure S2). The ability of the diffusive boundary to introduce non-BD-equilibrium DNA into gradient fractions can be modeled as a function of rotor geometry and the effect is more pronounced in vertical rotors relative to fixed angle rotors (Figure S2). Assuming sedimentation equilibrium, BD (ρ) can be directly related to the distance from axis of rotation (Birnie and Rickwood, 1978):

(4) $$x\;=\;\sqrt{(p\;-p_{m})\frac{2\beta^{{}^{\circ}}}{\omega^{2}}+r_{c}^{2}\;}$$

From this calculation, the location of DNA molecules in the centrifuge tube, both during centrifugation and fractionation, can be ascertained by using simple trigonometry along with knowledge of centrifuge tube dimensions and angle to the axis of rotation. A full description of the calculations along with an example can be found at https://github.com/nick-youngblut/SIPSim. The fraction of a taxon's DNA fragments that are in the boundary layer (*D*_*ti*_) is modeled as:

(5) $$D_{ti}=A_{ti}\gamma +\alpha$$

where *A*_*ti*_ is the pre-fractionation community relative abundance of taxon *t* in gradient *i*, γ is a weight parameter determining the contribution of *A*_*ti*_ to *A*_*b*_, and α is the baseline fraction DNA in *A*_*b*_.

Assimilation of the commonly used isotopes ^13^C and ^15^N into genomic DNA produces linear shifts in BD, with a maximum shift of 0.036 and 0.016 g ml^−1^, respectively (Birnie and Rickwood, 1978). Thus the shift in BD (ρ) can be modeled as:

(6) $$\rho_{13C}=I_{i,\max}A_{i}+\rho_{12C}$$

where *I*_*i,max*_ is the maximum possible BD shift if 100% atom excess for isotope *i, A* is the atom % excess of isotope *i*, and ρ_12*C*_ is the buoyant density at 0% atom excess. Note that the same formula applies to ^15^N.

### SIP data simulation framework overview

**Figure 1:**
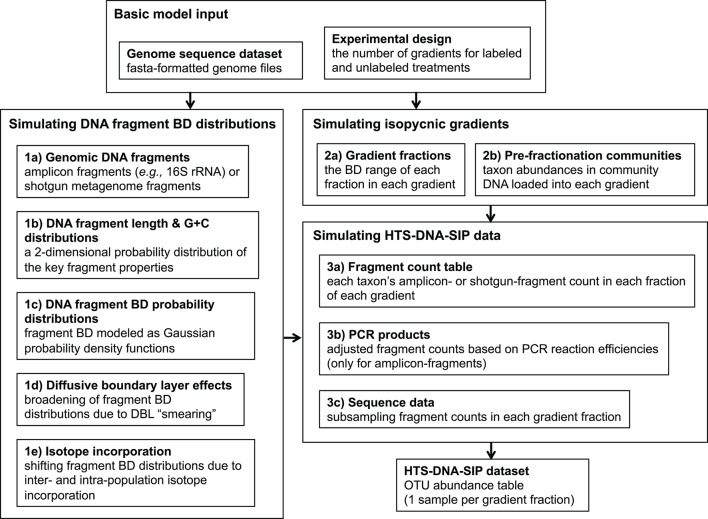
The SIPSim simulation workflow involves three major stages, which are broken down into multiple steps. Stage 1 involves generating a buoyant density distribution of gDNA fragments for each genome. Stage 2 involves simulating the isopycnic gradients for a particular experimental design. Stage 3 involves generating a DNA-SIP dataset based on the fragment BD value distributions simulated in Stage 1 along with the isopycnic gradient data generated in Stage 2. The output is a table (“DNA-SIP dataset”) of taxon relative abundances in each gradient fraction in each gradient. See section Methods for a more detailed description of the simulation workflow.

Based on the theory described above, our SIP data simulation framework simulates the distribution of gDNA fragments in isopycnic gradients at sedimentation equilibrium. Furthermore, it generates the DNA-SIP datasets obtained from fractionating isopycnic gradient(s) and performing high throughput sequencing on many of the gradient fractions. Our framework also implements all of the DNA-SIP analysis methods assessed in this study (Heavy-SIP, HR-SIP, MW-HR-SIP (see below), qSIP, and ΔBD) and evaluates their accuracy of identifying incorporators or quantifying BD shifts. An overview of our simulation framework is shown in Figure 1.

Our simulation framework is a modular collection of steps that can be grouped in workflow stages that are further broken down into steps (Figure 1). The input is a set of reference genomes in fasta format and a text file designating the experimental design, which includes the number of gradients for labeled treatments and unlabeled controls.

Stage 1 involves generating a BD distribution of gDNA fragments for each genome. Step 1a involves simulating the pool of gDNA fragments that is extracted from SIP incubation samples and then loaded into the isopycnic gradients. If amplicon sequence data (e.g., SSU rRNA) is to be generated, amplicons from only the fragments containing the PCR template (“amplicon-fragments”) are sequenced, while shotgun metagenomic sequencing can target all gDNA fragments (“shotgun-fragments”). If ≥1 PCR primer set is provided, amplicon-fragments are generated from genomic regions fully encompassing genome locations that produced amplicons by *in silico* PCR. Alternatively, shotgun-fragments are randomly generated from all possible genomic locations. The fragment size distribution is user-defined (Table S1).

As described in Equations (1, 3), the length and G + C content of a DNA fragment can be used to calculate a probability distribution of its location in the gradient, assuming sedimentation equilibrium. Step 1b uses the fragments simulated in Step 1a to generate a 2-dimensional Gaussian kernel density estimation (KDE) for each taxon, which describes the joint probability of obtaining fragments with a certain length and G + C content from that taxon. From this 2D-KDE, a large number of [length, G + C] vectors can be simulated efficiently for more precise estimations of the fragment BD distributions. Fragment BD distributions are calculated for each taxon in Step 1c by sampling [length, G + C] vectors from the 2D-KDE and calculating Gaussian distribution from each, where the mean is based on Eq. 1 and the standard deviation based on Equation (3). The collection of Gaussian distributions for all fragments for each taxon is integrated into a BD distribution for all fragments of a taxon with Monte Carlo error estimation, which involves sampling BD values from the collection of Gaussian distributions and estimating a probability density function (PDF) of the fragment BD distribution as a one-dimensional Gaussian KDE. The result is a list of KDEs, with each describing the probability of detecting the gDNA fragments of a taxon at any point along the isopycnic gradient. These fragment BD distributions are modified in steps 1d and 1e by adding diffusive boundary layer (DBL) effects (see section Theory Underlying the Simulation Framework) and isotope incorporation, respectively. The “smearing” due to DBL effects is modeled as a uniform distribution describing the increased fragment BD uncertainty, and this uncertainty is integrated into the fragment BD distributions by Monte Carlo error estimation as in Step 3b. The BD shift due to isotope incorporation is modeled in a similar manner, except BD uncertainty is a result of inter- and intra-population variation in the amount of isotope incorporated. Variation of isotope incorporation is modeled as a hierarchical set of mixture models (weighted sets of standard distributions; such as two Gaussians), where the parameters for intra-population mixture models that describe the amount of isotope incorporated by each individual are themselves defined by inter-population mixture models that describe how isotope incorporation varies among taxa.

Stage 2 involves simulating the isopycnic gradients for a particular experimental design. Step 2a involves simulating the BD range size of each fraction of each gradient. Sizes are drawn from a user-defined distribution. Step 2b involves simulating the relative abundance distribution of taxa in the gDNA pools loaded into each gradient (“pre-fractionation communities”). The abundance distribution of each pre-fractionation community is user-defined and can vary among gradients. Furthermore, the amount of taxa shared or rank-abundances permutated among communities (i.e., the beta-diversity) is user-defined.

Stage 3 involves generating a DNA-SIP dataset based on the fragment BD distributions simulated in Stage 1 along with the isopycnic gradient data generated in Stage 2. In Step 3a, an OTU (taxon) abundance table is generated by sampling from the fragment BD distributions of each taxon generated in Stage 1, with sampling depth determined by pre-fractionation community abundances simulated in Step 2b. The subsampled fragments are then binned into gradient fractions simulated in Step 2a. The resulting OTU table lists the number of gDNA fragments of each taxon in each gradient fraction in each gradient. If the simulated fragments are amplicons, then PCR amplification efficiency biases are simulated in Step 3b based on the PCR kinetic model described in Suzuki and Giovannoni (1996). The model assumes that efficiencies decrease as the product concentration increases due to an increased propensity of single stranded products to re-anneal to their homologous complements. Sequence data is simulated in Step 3c by subsampling from the table of fragment counts (the DNA fragment pool), which produces a final table (“DNA-SIP dataset”) of taxon relative abundances in each gradient fraction in each gradient.

### SIP data simulation framework parameters

Unless stated otherwise, we made the following assumptions for all simulations in this study. Community abundance distributions were simulated as lognormal distributions with a mean of 10 and a standard deviation of 2. All taxa were shared among communities, and no rank-abundances were permuted. The total number of fragments in each gradient was 1e^9^. Gradient fragment BD range sizes were sampled from a normal distribution, with a mean of 0.004 and a standard deviation of 0.0015. SSU rRNA amplicon-fragments were simulated using the V4-targeting 16S rRNA primers: 515F and 927R (5′-GTGYCAGCMGCMGCGGTRA-3′; 5′-CCGYC AATTYMTTTRAGTTT-3′), as used by Pepe-Ranney et al. (2016a). The amplicon-fragment size distribution was a left-skewed normal distribution with a mean of ~12 kb, which is similar to size distributions produced from common bead beating cell lysis methods (Kauffmann et al., 2004; Roh et al., 2006; Thakuria et al., 2008). A total of 1e^4^ amplicon-fragments were simulated per genome, which equated to >100X coverage for the genomic region of interest. Monte Carlo error estimation was conducted with 1e^5^ sampling replicates. Ultracentrifugation conditions were set as in Pepe-Ranney et al. (2016a), with a Beckman TLA-110 rotor spun at 55,000 rpm for 66 h at 20°C and an average density gradient 1.7 g ml^−1^. Inter-population variation in isotope incorporation was binary (either 0% or X% atom excess), and intra-population variation was set to zero. Two key parameters were estimated from empirical DNA-SIP data: the bandwidth (smoothing factor) for kernel density estimation, and the gamma parameter in Equation (5). See Table S1 for a full listing of simulation parameters.

### Implementing DNA-SIP analyses

The HR-SIP method was performed as described in Pepe-Ranney et al. (2016a,b). Briefly, we used a “heavy” BD window of 1.71–1.75 g ml^−1^, a sparsity cutoff of 0.25 (i.e., OTUs must be present in >25% of samples), a log_2_ fold change null threshold of 0.25, and a false discovery rate cutoff of 10%. ΔBD was determined as described by Pepe-Ranney et al. (2016a), with OTU abundances linearly interpolated across 20 evenly spaced values across the gradient BD range. Briefly, ΔBD is calculated as the difference in the center of mass of abundance distributions (i.e., the average BD weighted by relative abundance) between the labeled treatment and unlabeled control.

qSIP was conducted as described in Hungate et al. (2015), with 90% confidence intervals calculated from 1,000 bootstrap replicates. The variance among qPCR replicates was modeled based on the qPCR data provided in Table S2 of Hungate et al. (2015). Specifically, we found the qPCR count variance (σ^2^) to increase as a function of the mean (μ). The following polynomial regression was found to best describe this relationship and was used for simulating all qPCR count values:

(7) $$\sigma^{2}=5889+\mu +0.714\mu^{2}$$

where μ was set as the total number of simulated DNA fragments in the gradient fraction (designated in the OTU table from Step 4a).

A range of alternative analytical approaches to Heavy-SIP can be used to detect incorporators. We characterized four additional approaches to identifying labeled OTUs. Method 1 identifies as labeled any OTU that occurs in “heavy” fractions of the labeled gradient. Method 2 identifies as labeled any taxa present in the “heavy” fractions of the labeled treatment and absent from the “heavy” fractions of the control gradient. Method 3 identifies as labeled any taxa present in the “heavy” fractions of the labeled treatment and absent in the “light” fractions of the labeled treatment. Method 4 identifies as labeled any taxa present in the “heavy” fractions of the labeled treatment and absent from both the “heavy” fractions of the control and the “light” fractions of the labeled treatment. Of these four approaches, Method 1 provided the highest accuracy (Figure S6) and so this is the method that we used to represent “Heavy-SIP.”

We hypothesized that HR-SIP sensitivity could be improved by altering the “heavy” BD window (1.71–1.75 g ml^−1^) in which sequence composition is compared between treatment and control. We used SIPSim to evaluate a range of analytical approaches (data not shown) and found that the analysis of multiple windows (hereby called “MW-HR-SIP”) resulted in a significant improvement in sensitivity relative to HR-SIP. MW-HR-SIP evaluates sequence composition within BD windows of: 1.70–1.73, 1.72–1.75, 1.74–1.77 g ml^−1^ (Figure S3) while adjusting for multiple comparisons.

### Datasets

The genome dataset used to simulate genomic DNA fragments was obtained from Genbank (Benson et al., 2008). From a list of all bacterial genomes designated as “complete,” one representative was chosen per species in order to reduce the bias toward highly represented species. We found the dataset to contain a rather high proportion (~12%) of low G + C organisms (<30% G + C); most of which were obligate endosymbionts. We randomly sampled a subset of these low G + C genomes in order to reduce the proportion of low G + C organisms to just 1% of the genome dataset. The resulting dataset consisted of 1,147 bacterial genomes.

In order to simulate empirical data from Lueders et al. (2004), the genome sequences of *Methanosarcina barkeri* MS and *Methylobacterium extorquens* AM1 were downloaded from Genbank. Amplicon-fragments were simulated with the primers Ar109f (5′-ACKGCTCAGTAACACGT-3′), Ar915r (5′-GTGCTCCCCCGCCAATTCCT-3′), Ba519f (5′-CAGCMGCCGCGGTAANWC-3′), and Ba907r (5′-CCGTCAATTCMTTTRAGTT-3′). Atom % excess was assumed to be 100%, and isopycnic centrifugation conditions were simulated as specified in Lueders et al. (2004).

For model evaluation (see Supporting Results), we downloaded the genomes *Clostridium ljungdahlii* DSM 13528, *Escherichia coli* 1303, and *Streptomyces pratensis* ATCC 33331 from Genbank.

We compared the properties of DNA fragment BD distributions between simulated DNA-SIP data and empirical DNA-SIP data obtained from a soil community. The DNA-SIP dataset from Youngblut and colleagues consisted of SSU rRNA MiSeq sequences (V4 region) of ~24 fractions per gradient from 6 gradients of unlabeled controls. This dataset was generated using agricultural soils (15 g per sample) amended with a complex substrate mixture (3.6 mg C per g soil), and incubated aerobically at 50% water holding capacity and at room temperature. DNA was extracted following destructive sampling of replicates at days 1, 3, 6, 14, 30, and 48. DNA was subject to CsCl centrifugation (TLA110 Beckman rotor, 55,000 rpm, 66 h, 1.69 g ml^−1^ average gradient density) and fractionated (100 μl fractions) using methods which have previously been described in detail (Pepe-Ranney et al., 2016b). Since the use of these samples in the present context is limited to the analysis of DNA buoyant density distribution properties in the gradient, only control samples containing unlabeled DNA were examined. These data were subsampled to obtain a total richness equal to the 1,147 OTUs in our reference genome dataset. The sequence data is available from the NCBI under BioProject PRJNA382302.

### Software implementation

The SIP simulation framework was mostly written in Python v2.7.11, with some accompanying code written in C++ v4.9.2 and R v3.2.3 (R Core Team, 2016). MFEprimer v2.0 was used to perform *in silico* PCR (Qu et al., 2009). The software, along with documentation and examples, can be found at https://github.com/nick-youngblut/SIPSim. All genomes were downloaded from Genbank with the R package *genomes* v2.12.0 (Stubben, 2014), and all data analysis was conducted in R with the following packages: ggplot2 v2.1.0, dplyr v0.4.3, tidyr v 0.4.1, and cowplot v0.6.2.

Further methodological details are provided in the Supplementary Material.

## Results

### Model validation and parameter estimation

**Figure 2:**
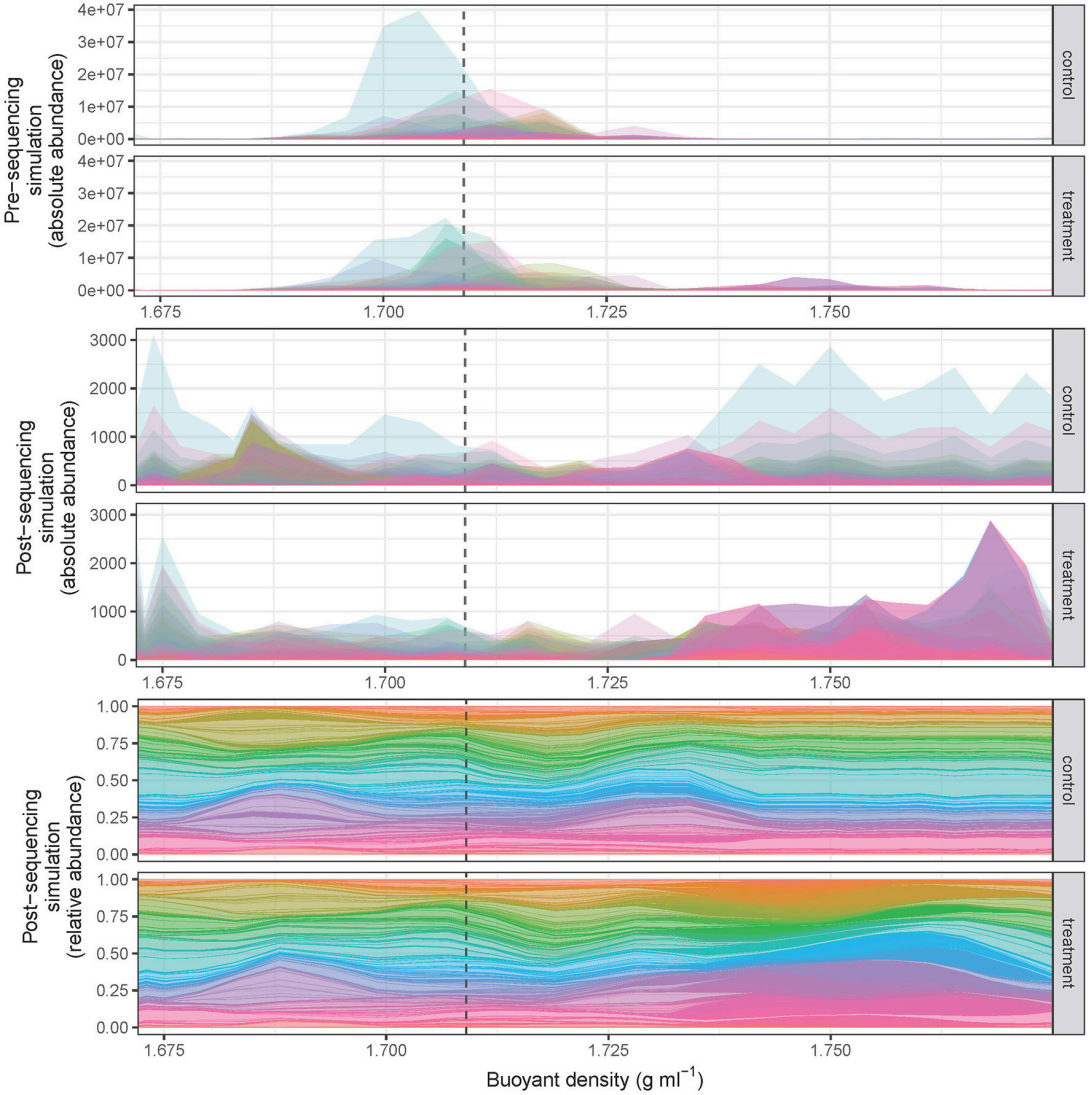
SIPSim output provides data that approximates results obtained from DNA-SIP experiments. The CsCl gradient BD distributions of diverse amplicon fragments (*n* = 1,147 taxa) are depicted such that the distribution of each taxon is represented by a different color. All taxa in the control had 0% atom excess ^13^C, while 10% of taxa in the treatment were randomly assigned 100% atom excess ^13^C. Most unlabeled amplicon fragments occur within the range of 1.69–1.72 g ml^−1^, while ^13^C-labeled taxa are shifted into higher BD fractions (pre-sequencing, top panels). During the process of high-throughput DNA sequencing amplicon fragments are randomly sampled from each fraction, and this sampling effect alters the shape of the fragment distributions observed in DNA-SIP experiments (post-sequencing, middle panel) relative to the actual distribution of DNA in the gradient (top panels). Typically, data from DNA-SIP experiments are transformed into relative abundance values (post-sequencing, bottom panel) prior to analysis. Identification of taxa that have incorporated isotope requires comparison of amplicon fragment relative abundance distributions in treatment relative to control gradients. The dashed vertical line is provided as a point of reference and designates the theoretical buoyant density of an unlabeled DNA fragment with 50% G + C (as modeled in Equation 1).

The SIPSim model starts with a set of user-designated genomes and user-designated experimental parameters (e.g., number of gradient fractions, desired community characteristics, desired isotopic labeling characteristics) as described (see section Methods and Supplementary Material). Briefly, the genomes are fragmented as would occur during DNA extraction, isotopic labeling is applied to some number of genomes as specified by the user, the BD distributions are determined for each DNA fragment and fragment collections are then binned into gradient fractions, fragments are sampled from each fraction as would occur during amplification and DNA sequencing of SSU rRNA genes, and then the relative abundance is calculated for each OTU (Figure 1, see also section Methods). The model produces results that are highly similar to those observed in empirical experiments, including the ability to detect DNA fragments throughout the density gradient (Figure 2).

The development of the simulation model was guided by established centrifugal theory and by comparison of simulated results to empirical data (as in section Methods and Supplementary Material). First, we performed a simple evaluation of model performance by recreating results from a prior DNA-SIP experiment with *Methanosarcina barkeri* MS and *Methylobacterium extorquens* AM1 (Lueders et al., 2004) (Figure S4). Simulated DNA distributions (both in terms of total DNA and SSU rRNA gene amplicon copies) significantly and strongly correlated with the empirical data for both taxa (*p* < 0.003 for all comparisons; see Table S2). In addition, the simulated SSU rRNA gene amplicon-fragment BD distributions were shifted 0.007 g ml^−1^ toward the middle of the BD gradient relative to the shotgun-fragments (“total DNA”), a phenomenon also observed in the empirical data. This central tendency for SSU rRNA amplicon-fragments reflects G + C conservation of the *rrn* operon, as previously described (Youngblut and Buckley, 2014).

**Figure 3:**
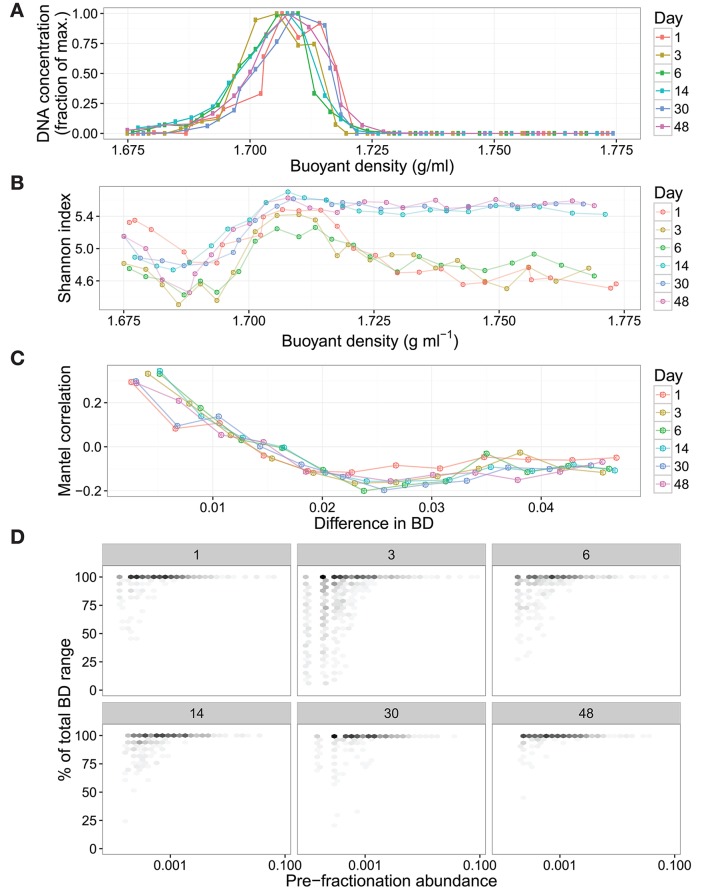
Empirical DNA-SIP data shows that unlabeled DNA is found widely within the gradient and that changes in beta-diversity can alter the composition of “heavy” fractions in the absence of isotopically labeled substrates. The DNA is from soil communities incubated for 1, 3, 6, 14, 30, or 48 days following the addition of an unlabeled nutrient mixture. SSU rRNA genes were amplified and sequenced from approximately 24 fractions from each gradient, these amplicons were used to identify the BD variance of amplicon fragments derived from discrete OTUs. The DNA concentration of each gradient fraction was measured using Picogreen assay **(A)**. These values are normalized to the maximum concentration within each gradient. The amplicon diversity within each gradient fraction was measured using the Shannon Index, showing that the diversity of heavy fractions differs between samples even in the absence of isotopic labeling **(B)**. The correlograms **(C)** reveal autocorrelation (measured with Mantel tests) between taxonomic similarity and fraction BD within each gradient. The variance in OTU BD is positively correlated with OTU pre-fractionation relative abundance, with highly abundant OTUs found throughout the CsCl gradient **(D)**. To improve clarity, single OTUs in **(D)** were binned into hexagons, with darker shading indicating more OTUs.

Next, we evaluated SIPSim results by comparing to empirical data. For this purpose SIPSim output was compared to results obtained with unlabeled DNA from soil (see section Datasets). The empirical data was derived from an experiment in which an unlabeled nutrient mixture was added to soil and DNA was extracted at 1, 3, 6, 14, 30, and 48 days. These six DNA samples were equilibrated in CsCl gradients, fractionated by BD, and SSU rRNA gene amplicons were sequenced for ~24 fractions from each gradient. The simulation run included 1,147 microbial genomes (see section Methods), hence the soil data was resampled to 1,147 OTUs in order to standardize the richness of the simulated and empirical data. The empirical results reveal several interesting features about the distribution of DNA in CsCl gradients. First, we observed that variance in DNA fragment BD is positively correlated with its relative abundance and that most taxa with relative abundances >0.1% are detected in all CsCl gradient fractions (Figure 3). Second, we observed that taxonomic similarity is auto-correlated with fraction BD, so that fractions of similar BD have similar nucleic acid composition (Figure 3). Finally, we observed that changes in community composition caused dramatic shifts in the sequence composition of “heavy fractions” even in the absence of isotopic labeling (Figure 3). We applied three different analytical approaches to assess similarity between empirical and simulated data: correlation in Shannon Diversity with respect to fraction BD, correlation in Jaccard dissimilarity with respect to fraction BD, and correlation in OTU BD range with respect to taxon relative abundance. We found that variance between simulated and empirical results was significantly less than the variance observed between replicate empirical samples (Figure S5). Furthermore, we used these comparisons between simulated and empirical results (Figure S5) to optimize parameter values for use in the SIPSim model (Table S1, as described in Supplementary Material).

### The influence of isotope incorporation on DNA-SIP accuracy

We hypothesized that both the number of taxa that incorporate isotope and the atom % excess isotope incorporation per taxon would substantially affect the accuracy of DNA-SIP methods. To test these predictions, we simulated DNA-SIP datasets for both ^13^C-labeled samples and unlabeled controls (3 replicates of each), while varying both the number of incorporators (1, 5, 10, 25, or 50% of taxa) and the atom % excess isotope incorporation for each taxon (0, 15, 25, 50, 75, or 100 atom % excess ^13^C). Taxa in the control were always set to 0% atom excess isotope incorporation. Each simulation was replicated 10 times, with differing taxa randomly designated as incorporators in each replicate. We evaluated 7 methods used to analyze DNA-SIP data including: HR-SIP, qSIP, 4 different approaches to “Heavy-SIP,” and MW-HR-SIP. Heavy-SIP includes a family of approaches (see section Methods) in which incorporators are identified on the basis of presence-absence in heavy and/or light fractions (Figure S6).

**Figure 4:**
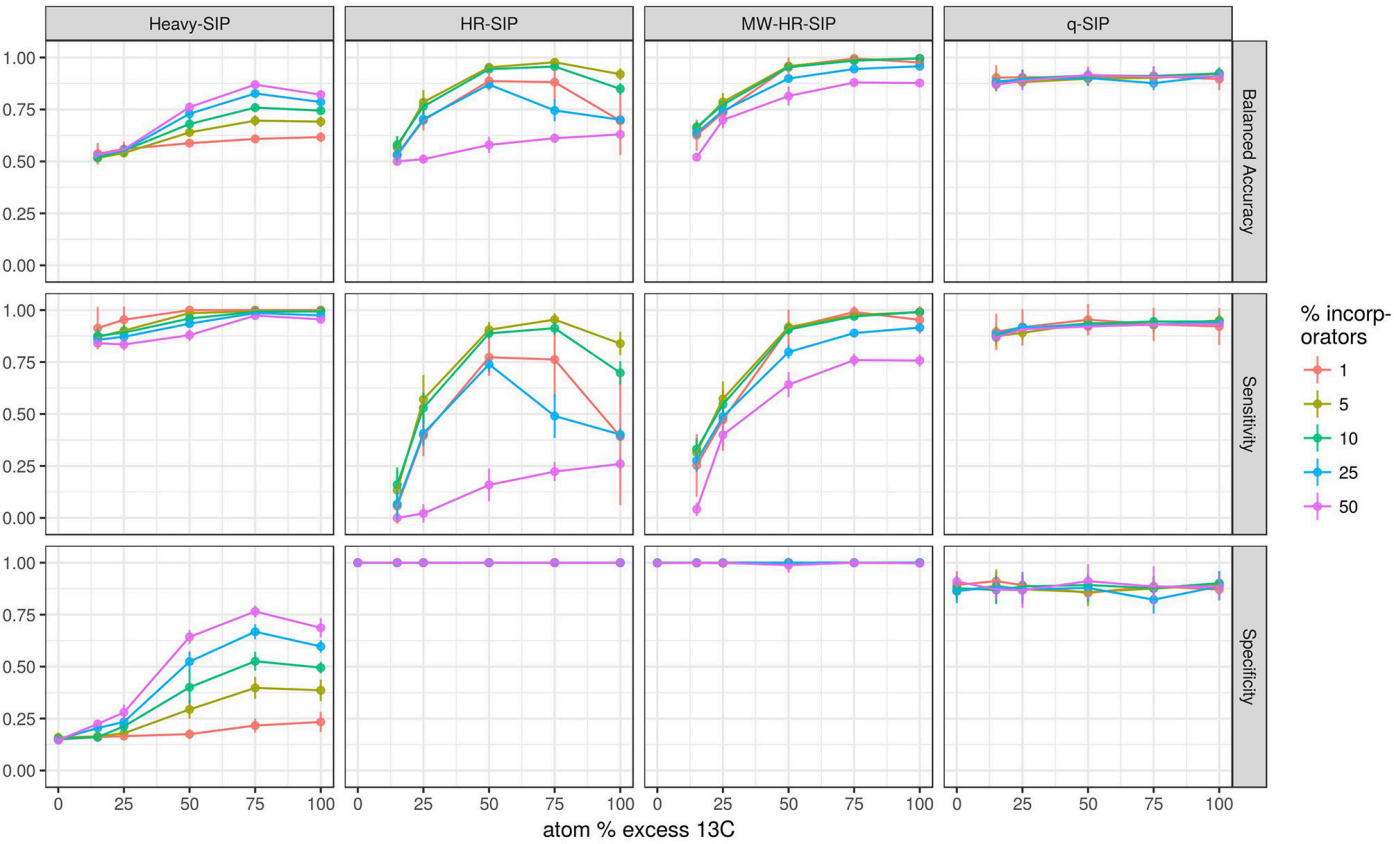
SIPSim predicts that DNA-SIP methods vary in accuracy depending on the ^13^C atom % excess of DNA and the number of taxa that incorporate isotope. Points and bars represent means and standard deviations, respectively (*n* = 10 simulations). Specificity indicates the fraction of true negatives that are identified correctly. Sensitivity indicates the fraction of labeled taxa (true positives) identified correctly. Balanced accuracy is the product of specificity and sensitivity. The *x*-axis indicates the amount of ^13^C isotope present in taxa that are labeled, and different colors are used to indicate the percentage of taxa that have incorporated ^13^C as indicated by the legend. Heavy-SIP identifies incorporators solely based on OTU presence in “heavy” gradient fractions, and this approach is shown because it has better balanced accuracy than any of the other Heavy-SIP approaches that were analyzed (see Figure S6).

The model predicts that both the number of incorporators and the amount of isotope incorporated affected accuracy (Figure 4). However, the predicted effect of these parameters on specificity and sensitivity varied depending on the analytical method (Figure 4). Specificity is the proportion of true negatives observed out of all true negatives expected, and so specificity declines in direct relation to an increase in the number of false positives. For example, a specificity of 0.8 would generate 200 false positives in a sample of 1,000 unlabeled taxa. Specificity, as measured across a wide range in parameters, was predicted to be highest for MW-HR-SIP (1.00 ± 0; ave. ± s.d.) and HR-SIP (1.00 ± 0), substantially lower for qSIP (0.88 ± 0.06), and very low for Heavy-SIP (0.28 ± 0.16) (Figure 4).

Sensitivity is the fraction of true positives observed out of all true positives expected. For example, a sensitivity of 0.7 means that a method failed to detect 30% of the incorporators present. Both qSIP and Heavy-SIP are predicted to have relatively high sensitivity (median values of 0.91 and 0.93, respectively), and the sensitivity of these methods was largely insensitive to the atom % excess of DNA and the number of incorporators (Figure 4). In contrast, the sensitivities of both HR-SIP and MW-HR-SIP were predicted to be highly responsive to the atom % excess of DNA, and the number of incorporators (Figure 4). For these methods, sensitivity is predicted to decline in proportion to the atom % excess ^13^C label in DNA.

Balanced accuracy is calculated as the mean of specificity and sensitivity. The model predicts a tradeoff in balanced accuracy in relation to the atom % excess ^13^C of DNA. MW-HR-SIP had the highest predicted accuracy of any of the 7 methods tested when % atom excess ^13^C exceeded 50%, but qSIP has higher accuracy at lower levels of isotope incorporation (Figure 4). This tradeoff in balanced accuracy resulted from a difference in the tolerance for false positives. For example, MW-HR-SIP produced nearly zero false positives but as a result of its high specificity, it lost sensitivity at lower levels of isotope incorporation. In contrast, qSIP detected labeled taxa across a wider range of isotope incorporation, but it did so at the cost of a large number of false positives.

### The influence of experimental parameters on DNA-SIP accuracy

**Figure 5:**
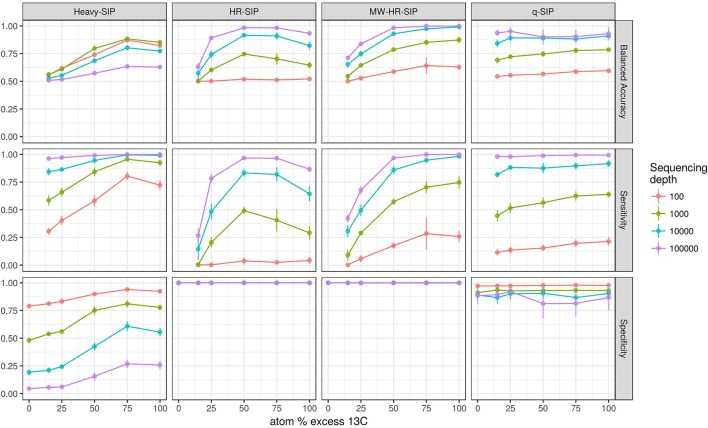
SIPSim predicts that DNA-SIP methods vary in accuracy depending on the number of sequences analyzed per gradient fraction. Points and bars represent means and standard deviations, respectively (*n* = 10 simulations). Specificity indicates the fraction of true negatives that are identified correctly. Sensitivity indicates the fraction of labeled taxa (true positives) identified correctly. Balanced accuracy is the product of specificity and sensitivity. The *x*-axis indicates the amount of ^13^C isotope present in taxa that are labeled, and different colors are used to indicate the average number of sequences determined per gradient fraction as described by the legend.

**Figure 6:**
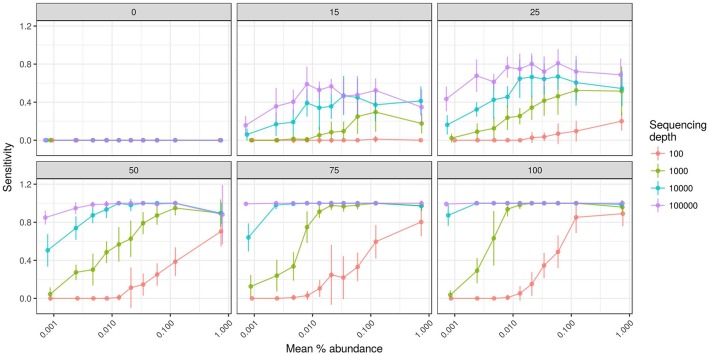
SIPSim predicts that the sensitivity of detecting OTUs that have low relative abundance and low atom % ^13^C enrichment increases with added sequencing effort per gradient fraction. Each panel provides results from simulations conducted at different levels of atom % ^13^C enrichment as indicated (0, 15, 25, 50, 75, and 100 atom % ^13^C), incorporators were identified using MW-HR-SIP, and sensitivity was assessed by binning OTUs into 10 different abundance classes. Sensitivity indicates the fraction of labeled taxa (true positives) identified correctly. The *x*-axis indicates the mean relative abundance of the taxa being evaluated, and different colors are used to indicate the average number of sequences determined per gradient fraction as described by the legend. Points and bars represent means and standard deviations, respectively (*n* = 10 simulations).

We also evaluated the effects of sequencing effort and fraction size on DNA-SIP accuracy because SIP experiments often vary in the number of fractions analyzed per gradient and the number of sequences analyzed per fraction. The model shows that sequencing effort has different consequences for method specificity and sensitivity. We found that the specificities of HR-SIP and MW-HR-SIP are predicted to be independent of sequencing effort, while those of Heavy-SIP and qSIP actually declined with sequencing effort (Figure 5). The predicted decrease in specificity for Heavy-SIP and qSIP indicates that the rate of false discovery for these methods increases in proportion to the number of sequences analyzed, while the rate of false discovery in HR-SIP and MW-HR-SIP is unaffected by the number of sequences analyzed. In contrast, we found that method sensitivity improved with sequencing effort for all analytical methods (Figure 5). This predicted increase in sensitivity is caused by an increase in statistical power caused by sampling more sequences from each gradient fraction. We further show that improvements in sensitivity are predicted to be greatest for taxa present at low relative abundance (Figure 6). For example, with MW-HR-SIP the sensitivity of detection for a 50% atom ^13^C enriched OTU present at 0.001 relative abundance is predicted to be nearly zero if less than 1,000 sequences are analyzed per gradient fraction, but sensitivity improves dramatically as sequencing depth increases (Figure 6).

SIP methods also will often vary in the number of fractions collected per gradient, and so we evaluated the effect of fraction size on DNA-SIP accuracy. The model predicts that the use of smaller fractions (i.e., collecting more fractions per gradient) tends to improve sensitivity and overall accuracy for HR-SIP, MW-HR-SIP, and qSIP, though the effects are modest (Figure S7). In contrast, Heavy-SIP is predicted to improve in accuracy when larger fractions are used, though this effect is also somewhat modest (Figure S7).

### The influence of community variation on DNA-SIP accuracy

All DNA-SIP analyses rely upon comparisons made between isotopically enriched experimental treatments and their corresponding unlabeled controls. In real SIP experiments, the composition of replicate post incubation communities are likely to vary somewhat as a result of sample heterogeneity and incubation effects. However, the simulations described above assume random sampling from identical pre-fractionation (post-incubation) community structures. We hypothesized that an increase in variation in community composition between treatment and control samples would decrease the accuracy of DNA-SIP analyses. To test this hypothesis, we generated simulations in which isotope incorporation was held constant (50 atom % excess ^13^C; 10% of OTUs are incorporators) but beta-diversity was varied among 3 replicate treatment and 3 replicate control samples. We varied beta-diversity in two ways: (i) using permutation to vary the rank abundance of a fixed proportion of community members and (ii) varying the proportion of taxa shared between communities. For each simulation scenario, we calculated the mean Bray-Curtis distance among communities in order to provide a real-world metric for gauging the potential accuracy of actual DNA-SIP experiments.

**Figure 7:**
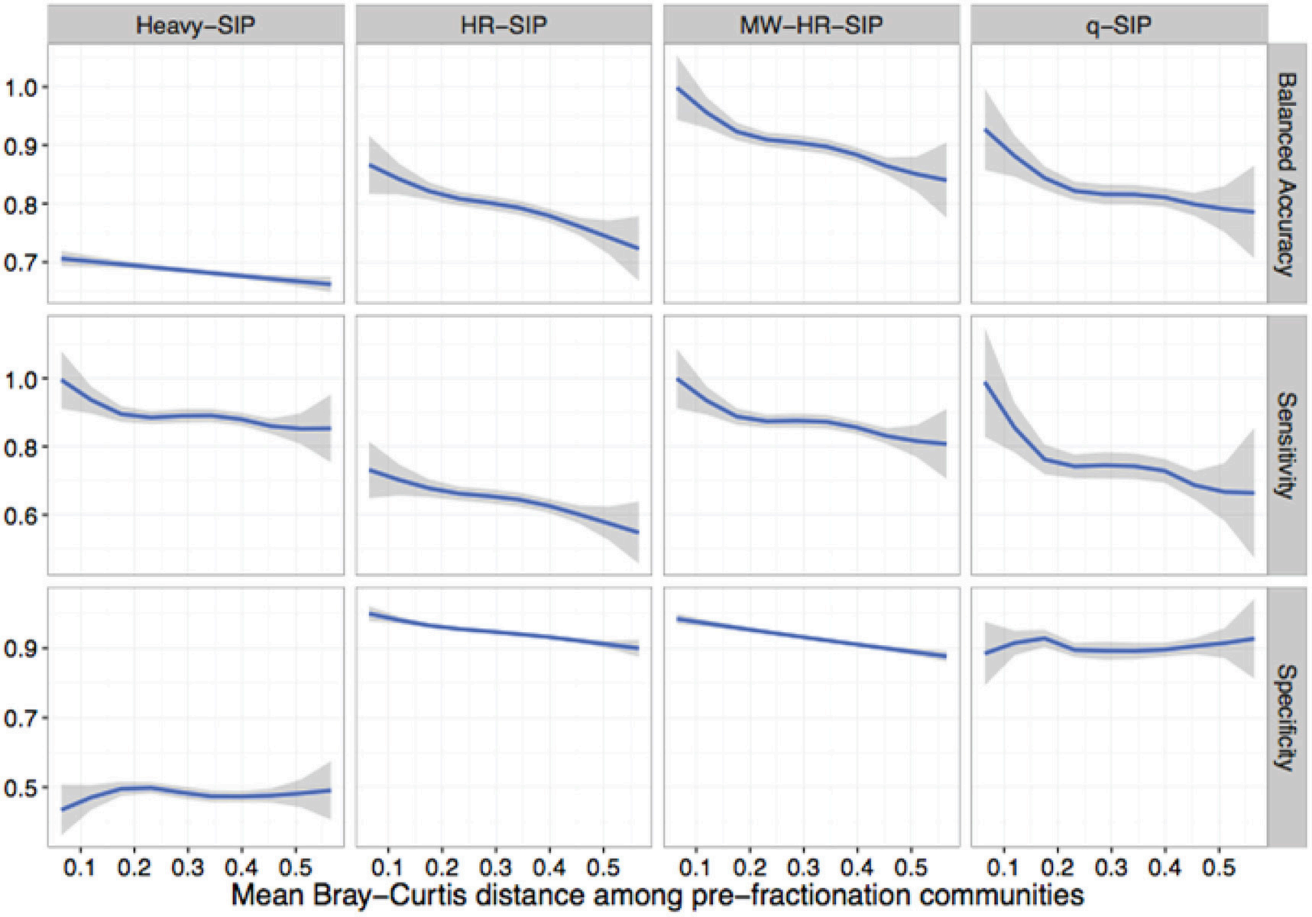
SIPSim predicts that DNA-SIP methods differ in their sensitivity to community dissimilarity between replicate samples. Beta diversity, expressed as Bray-Curtis dissimilarity, was varied between simulated replicates (3 replicates each for ^12^C-control and ^13^C-treatment gradients) to determine the effect that community dissimilarity between replicates has on method accuracy. Variation in beta diversity was simulated by systematically varying two parameters: the percent of taxa shared between replicate samples (80, 85, 90, 95, or 100%) and the percent of taxa whose rank abundances that were permuted (0, 5, 10, 15, or 20%), with 10 simulation replicates for each parameter set. The blue lines are LOESS curves fit to accuracy values for all simulations (*n* = 250), and the gray regions represent 99% confidence intervals. For all simulations, 10% of the community were incorporators (50% atom excess ^13^C).

The model predicts that increased beta-diversity among samples impacts the accuracy of DNA-SIP methods (Figure 7). The model predicts that accuracy is impacted more by the number of taxa shared between samples than by differences in taxon abundance (Figure S8). The sensitivity of all methods is predicted to decline as beta-diversity increases, falling from approximately 0.9 for both qSIP and MW-HR-SIP when samples shared 100% of their OTUs to 0.64 and 0.7 for qSIP and MW-HR-SIP, respectively, when samples shared only 80% of their OTUs (Figure S8). Increasing the beta-diversity between samples was predicted to have little effect on the specificity of qSIP but diminished the specificity of HR-SIP and MW-HR-SIP (Figure 7). MW-HR-SIP was predicted to have greater balanced accuracy than qSIP as the Bray-Curtis distance between treatment and control samples increased from of 0.0 to 0.4, but at higher levels of distance the two methods performed with similar accuracy (Figure 7). Regardless, it is clear that sample-to-sample variation has an overall negative impact on DNA-SIP accuracy, and this result emphasizes the importance of minimizing experimental variation between unlabeled controls and labeled treatments in SIP experiments.

### Using DNA-SIP data to quantify atom % excess

So far, we have focused on the accuracy of DNA-SIP methods with respect to the identification of taxa that incorporate isotope into their DNA. However, changes in DNA BD can also be used to quantify the isotope enrichment of DNA from particular taxa. Two approaches have been used to evaluate isotope enrichment from DNA-SIP data: qSIP and ΔBD, with the latter being a complementary analysis to HR-SIP (Pepe-Ranney et al., 2016a). Both ΔBD and qSIP derive quantitative estimates from measuring taxon BD shifts (and thus atom % excess) in the labeled treatment gradient(s) vs. their unlabeled counterparts. The ΔBD method attempts to measure the extent of the BD shift directly from the compositional sequence data, while qSIP utilizes relative abundances transformed by qPCR counts of total SSU rRNA copies. Therefore, ΔBD accuracy likely suffers from compositional effects inherent to HTS datasets, while qSIP accuracy is dependent on qPCR accuracy and variation.

**Figure 8:**
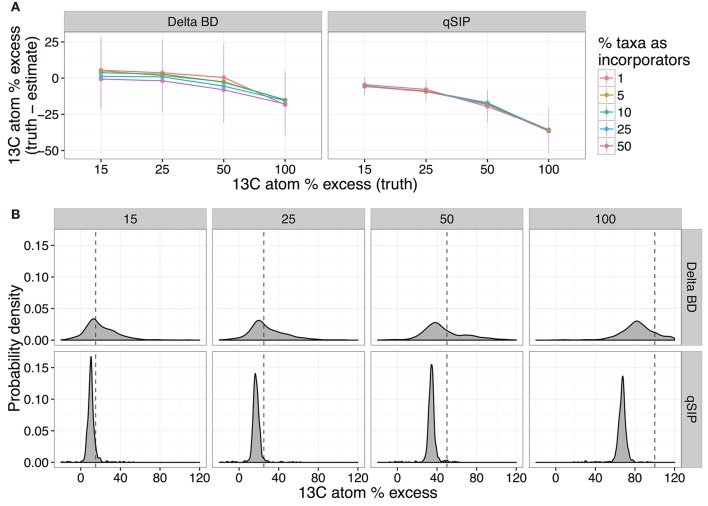
SIPSim predicts that ΔBD and qSIP vary in their accuracy at estimating ^13^C atom % excess of labeled DNA fragments. The accuracy of both methods declines as the amount of ^13^C in DNA increases **(A)**, but accuracy is not affected by the percent of taxa that are labeled; values indicate the mean and standard deviation (*n* = 10 simulations). Probability density plots indicate that estimates of ^13^C atom % excess made using Δ*BD* have greater variance than those made using *qSIP*, but both estimates systematically underestimate levels of isotope incorporation **(B)**. Each vertical pair of panels indicates the probability density for estimates made across different levels of isotope incorporation (15, 25, 50, and 100 atom % excess), and the dashed line indicates the actual level of isotopic enrichment. For the calculation of probability density, 10% of taxa were labeled using the level of enrichment indicated in each panel.

We assessed the quantification accuracy of both methods using the simulations described previously, where either the amount of isotope incorporation or sample beta-diversity was varied. The model predicts that ΔBD produced estimates of isotope incorporation that are closer on average to the true value compared to qSIP, but ΔBD values had much higher variance than qSIP estimates (Figure 8). Furthermore, the predicted variance in ΔBD atom % excess ^13^C estimates increased substantially with even moderate increases in beta-diversity between samples, while the qSIP estimations were largely invariant across the simulation parameter space (Figure S9A). Both methods are predicted to consistently misestimate true ^13^C atom % excess, though the effect was greater for qSIP, with qSIP underestimating ^13^C atom % excess by 30.2–39.2% for fully labeled DNA (Figure 8B).

We further investigated several factors to determine whether they impact the estimation of ^13^C atom % excess from DNA-SIP data. The model predicts that the size of gradient fractions (Figure S10) and the depth of sequencing per fraction (Figure S11) had little impact on estimation of ^13^C atom % excess for OTUs. Furthermore, the model predicts that combining techniques, by using MW-HR-SIP to first identify labeled taxa and then using qSIP to calculate the ^13^C atom % excess of OTUs, reduces the variance of ^13^C atom % excess estimates but does not correct for the systematic underestimation of ^13^C atom % excess (Figures S10, S11). Finally, we attempted to determine why qSIP systematically underestimates the ^13^C atom % excess of OTUs. We find that the degree to which qSIP underestimates ^13^C atom % excess is predicted to increase in proportion to the actual ^13^C atom % excess of each OTU (Figure S12). This outcome could be explained by qSIP's use of weighted averaging in estimating ^13^C atom % excess. The use of weighted averages to estimate the BD of each OTU assumes a roughly Gaussian BD distribution, which we show not to be the case (Figure S1). The BD distributions of “heavy” DNA fragments will be left skewed in a CsCl gradient and the weighted average of a left skewed distribution will cause systematic underestimation of ^13^C atom % excess with the degree of underestimation increasing in proportion to the BD of the DNA, as observed.

## Discussion

Our simulation framework (SIPSim) provides a tractable platform for evaluating the accuracy of DNA-SIP methods and for developing new methods to analyze DNA-SIP data. Given the laborious nature of DNA-SIP experiments, it is impractical to use empirical analyses with mock communities to evaluate the range of parameter values that can be investigated readily through simulation (e.g., we simulated >1,000 SIP experiments in this effort). In addition, both the physics of density gradient centrifugation and the physical properties of genomic DNA are well established, making the simulation of DNA-SIP data both tractable and reliable. Without rigorous assessment of DNA-SIP methods, it is difficult to determine the likelihood of false negatives (Type II error) and false positives (Type I error) across the wide range of experimental conditions in which DNA-SIP has been employed in the literature. Issues of Type I and Type II statistical error are compounded by the nature of high-throughput sequencing data, where it is necessary to make many thousands of comparisons to identify OTUs that change in response to treatment. This multiple comparison problem has major implications for statistical power and the likelihood of false detection (Paulson et al., 2013). We have used SIPSim to test the effects of multiple parameters on the accuracy of current methods for analyzing DNA-SIP data.

Different approaches for detecting isotope incorporators result in substantial differences in sensitivity and specificity. The model predicts that both qSIP and MW-HR-SIP are superior to several different “Heavy-SIP” approaches (Figures 4, 7 and Figure S6). The qSIP method is predicted to have high sensitivity but low specificity, resulting in a large number of false positives (8 ± 0.3 to 15 ± 0.7% of the unlabeled taxa which were evaluated were misidentified as labeled; Figures 4, 7). In contrast, MW-HR-SIP is predicted to have high specificity and negligible false positives (Figures 4, 7), but had lower sensitivity (more false negatives). This tradeoff between specificity and sensitivity can be contextualized by considering a community that contains 1,100 taxa, 55 of which are isotopically labeled. If these 55 taxa are labeled at 50% atom excess ^13^C, both methods do a good job of detecting labeled taxa (true positives: MW-HR-SIP, 51 ± 2; qSIP, 50 ± 2), but qSIP detects many false positives (false positives: MW-HR-SIP, 0 ± 1; qSIP, 126 ± 8). If these 55 taxa are instead labeled at 25% atom excess ^13^C then MW-HR-SIP detects fewer labeled taxa (true positives: MW-HR-SIP, 33 ± 3; qSIP, 50 ± 2), but qSIP continues to detect many false positives (false positives: MW-HR-SIP, 1 ± 0; qSIP, 122 ± 8). In these examples, >97% of the taxa identified by MW-HR-SIP are truly labeled, while only about 29% of those identified by qSIP are actually labeled (note that this example contextualizes the number of false positives relative to the sum of true and false positives, while specificity is formally defined as the number of true negatives observed relative to true negatives expected). It is possible that the low specificity of qSIP could be caused by the fact that this method employs 90% confidence intervals to identify as ^13^C-labeled those taxa that have a large BD increase in response to ^13^C-labeling. It is possible that modification of qSIP to employ 99% confidence intervals could result in an improvement of specificity, however, such a change would also certainly diminish sensitivity and so the overall impact on balanced accuracy is difficult to predict at this time. Further, improvement of DNA-SIP analyses should be facilitated by use of the SIPSim framework.

In regards to methods used to quantify the atom % excess of individual taxa from DNA-SIP data, we found that the utility of qSIP or ΔBD is predicted to vary depending on the hypothesis being evaluated. ΔBD produced more accurate estimates of mean ^13^C atom % excess than qSIP (Figure 8 and Figure S8), and so this approach may be suitable when seeking to make relative comparisons in the degree of labeling between large groups of taxa (as described in Pepe-Ranney et al., 2016b). However, the high variability of this approach causes ΔBD to be unreliable in determining differences in atom % excess ^13^C at the scale of individual OTUs. Alternatively, qSIP is predicted to produce much more stable estimates of atom % excess ^13^C among individual taxa, but the method is predicted to produce systematic underestimates of isotope incorporation. We hypothesize that qSIP is underestimating atom % excess ^13^C because it uses weighted averaging to calculate the BD of each OTU. We expect that a statistical approach less sensitive to the violations of normality that occur in CsCl gradients may improve the ability of qSIP to accurately estimate atom % excess ^13^C values.

The SIPSim framework makes it possible to evaluate hypothetical outcomes of DNA-SIP experiments before they are performed and to evaluate the accuracy of DNA-SIP data analysis methods. For brevity, we have only focused on a few key variables that could affect the accuracy of DNA-SIP methods. However, SIPSim can also be used to assess the accuracy of DNA-SIP methods across a range of possible real-world scenarios. For instance, spatial or population-level heterogeneity could result in taxa that are not homogeneously labeled (Lennon and Jones, 2011). Such systematic heterogeneity in labeling would manifest as “split” (bimodal or multimodal) distributions of DNA fragments in an isopycnic gradient. It would be challenging to evaluate such scenarios empirically, but SIPSim can be readily used to evaluate a range of such scenarios. SIPSim also provides a toolkit for developing and improving analytical methods used in DNA-SIP experiments.

## Conclusion

With our newly developed simulation toolset, we determined that MW-HR-SIP is predicted to have the lowest false positive rate of all methods tested for analyzing DNA-SIP data. The use of MW-HR-SIP resulted in a negligible number of false positives and its ability to detect true positives varied in relation to OTU isotopic enrichment and relative abundance. Sensitivity is predicted to improve with increases in sequencing effort. Generally, SIPSim predicts that the specificities of all DNA-SIP methods decline with increased beta-diversity among replicate samples. Thus, given that accuracy is predicted to decline most rapidly between a mean Bray-Curtis distance of 0 and 0.2 for all methods evaluated (Figure 7), we recommend that researchers strive for mean Bray-Curtis distances of <0.2 among replicate samples used in SIP experiments (i.e., between treatments and their corresponding controls).

## Author contributions

NY developed the code for SIPSim, performed and analyzed all simulations, developed the figures and wrote the manuscript. SB developed the approach to modeling diffusive boundary layers in equilibrium density gradients. DB conceived the simulation approach, supervised and directed the research, and edited the manuscript.

### Conflict of interest statement

The authors declare that the research was conducted in the absence of any commercial or financial relationships that could be construed as a potential conflict of interest.
