# Supplementary material for: SIPSim: A Modeling Toolkit to Predict Accuracy and Aid Design of DNA-SIP Experiments
Source: Front Microbiol. 2018 Mar 28;9:570. doi: 10.3389/fmicb.2018.00570 (PMC5882788; doi:10.3389/fmicb.2018.00570)
Supplement: Supplementary file 1 [file Presentation1.PDF]

Supplementary Material

Evaluating the accuracy of DNA stable isotope probing

Nicholas D. Youngblut, Samuel E. Barnett, and Daniel H. Buckley*

***Correspondence:** Daniel H Buckley, dbuckley@cornell.edu

1 Supplementary Methods

1.1 Comparing empirical to simulated data and estimation of model parameters

DNA-SIP simulation output was compared to empirical data to determine the degree to which model outputs matched reality. Furthermore, we estimated two key model parameters based on the empirical DNA-SIP data.

The empirical DNA-SIP dataset consisted of SSU rRNA gene sequences from six CsCl gradients, which represented the unlabeled control samples from a larger DNA-SIP experiment performed on a soil microbial community (see *Methods*). Ideally, we could map SSU rRNA sequences from the genomes found in soil to identical bacterial genomes available in public databases. However, genome composition can vary dramatically even for taxa that have identical SSU rRNA gene sequences. Since the genome sequences of taxa in the empirical DNA-SIP dataset could not be confidently assigned to genomes in existing databases based solely on the partial SSU rRNA sequence identity, a direct mapping of taxa (and their genomes) between the empirical and simulated datasets was not possible.

We therefore took a different approach of comparing these datasets by using metrics that quantify variation in DNA fragment BD distributions. For example, the Shannon index is sensitive to a change in richness and this value will remain constant across the gradient if DNA fragments have wide BD distributions. However, if BD distributions are narrow Shannon will drop towards the end of the gradient (Figure 3). We also measured the degree of autocorrelation in community composition (as measured by Jaccard dissimilarity) across gradient fractions (Figure 3). Autocorrelation will be high if DNA fragment BD distributions are wide and it will decrease as these distributions narrow. Finally, we measured the BD range of each taxon (*i.e.* the range in BD across which a taxon was observed) as a function of each taxon's pre-fractionation abundance in the community (Figure 3).

To determine whether these metrics were effective at quantifying variation in DNA fragment BD distributions, we performed simulations in which we manipulated the properties of fragment BD distributions by systematically varying two community parameters: the variance of each Gaussian ("sigma") and the variance among means of each Gaussian ("optima"). Each simulation consisted of 1200 Gaussian distributions (each distribution

representing amplicon fragments from a single taxon) spanning a gradient comprising 30 fractions (Figure S13). The Shannon index was calculated for each fraction in each simulation, and all pairwise Pearson correlation coefficients were calculated between simulations (Figure S14). For the calculation of each correlogram, we first produced distance matrices of Jaccard dissimilarities among all fractions in each simulation. Correlograms generated from the Jaccard values were compared between simulations via Pearson correlation coefficients (Figure S15). Overall, we found that BD distributions having similar properties (similar sigma and optima) produced high correlation values for both Shannon index and Jaccard dissimilarity. This means that these metrics are effective at quantifying similarity in the properties of BD distributions between gradients. The R package *vegan* v2.2 was used to calculate the Shannon index, Jaccard dissimilarities, and the correlograms. The R package *coenocliner* v0.2 was used to simulate Gaussian taxon abundance distributions across a density gradient for an initial assessment of these three metrics (Simpson, 2015).

Using these three metrics, we compared the empirical dataset to simulation results (Figure S5). We varied two parameters across the simulations: the bandwidth (smoothing) factor for kernel density estimation (KDE), and also the weighting parameter (γ) for setting the relative amount of DNA fragments contributing the to DBL effects (see Eq. 5). Across much of the parameter space tested, we observed stronger correlations between simulated and empirical results than between independent replicates from the empirical data (Figure S5). This means that variation between empirical and simulated datasets was often less than the true variation among the 6 gradients in the empirical dataset (Figures 3 and Figure S5). In general, weighting factors (γ) of 0.4-0.8 generally produced the strongest correlations of Shannon Index values, while bandwidths of 0.6-0.8 produced the strongest correlations between correlogram values (Figure S5). Based on all three of these metrics, we selected a bandwidth of 0.8 and a γ of 0.5 for all other DNA-SIP dataset simulations (Table S1).

1.2 Software implementation

The SIP simulation framework was mostly written in Python v2.7.11, with some accompanying code written in C++ v4.9.2 and R v3.2.3 (R Core Team 2016). Python package dependencies include: numpy, scipy, docopt, cython, dill, and pathos (Jones *et al.* 2016; McKerns et al. 2011). The R package dependencies include: ggplot2, dplyr, tidyr, docopt, phyloseq, and caret (Wickham 2009; McMurdie and Holmes 2013; Wickham and Francois 2016; Wickham 2016). Most steps of the simulation framework can be run in parallel if multiple processors are available. The software is freely available on GitHub at https://github.com/nick-youngblut/SIPSim.

2 Supporting Results

2.1 Model evaluation

We employed multiple methods to evaluate whether simulation output matched our theoretical predictions. First, we simulated SSU rRNA amplicon-fragments from the genomes of *Clostridium ljungdahlii* DSM 13528, *Escherichia coli* 1303, and *Streptomyces pratensis* ATCC 33331, which have genome G + C contents of 31.1, 50.7, and 71.1, respectively. Three different fragment length distributions were simulated, which represent either *i*) highly sheared DNA, *ii*) only high molecular weight DNA, *iii*) a typical DNA length distribution from bead-beating cell-lysis methods (Figure S16). As expected, genomic G + C content dictated the mean of each fragment BD distribution, and fragment length had a substantial influence on the variance of each distribution (Figure S16). Modeling fragments in the gradient as homogeneous bands of DNA molecules, each with a Gaussian shape, substantially increased the BD distribution variance relative to modeling fragment BD as a discrete value based solely on G + C content (Figure S17). This increase in BD distribution variance was least pronounced for high molecular weight fragments, which was expected given that the variance of each individual Gaussian distribution is inversely related to fragment length (Eq. 3). Simulating diffusive boundary layer (DBL) effects due to using of a fixed-angle rotor (see *Experimental Procedures*) did not substantially alter the BD distribution except for considerably increasing the probability density at the tails of the distributions (Figure S1).

In order to assess the generality of these assessments across diverse taxa, we simulated amplicon-fragments from all genomes in our bacterial genome dataset (n = 1147) and plotted fragment BD distributions for each BD modeling approach (Figure S18). For this analysis, we focused on the "typical" left-skewed normal distribution to model fragment lengths. Across all BD distributions for each genome, the median BD varied from ~1.695-1.173 g ml^{-1} regardless of the method for modeling BD distributions. This finding was previously observed in Youngblut and Buckley (2014), where fragment BD was modeled as in Eq. 1 (Youngblut and Buckley 2014). Modeling homogenous DNA fragments as Gaussian bands expanded the 5-95 % percentile range only slightly relative to discrete calculations of BD, but the 0-100 % percentile range expanded substantially from ~0.015 to ~0.055 g ml^{-1}. Incorporating DBL effects into the model enhanced the spread of both the 5-95% percentile range, but more importantly, it made all 0-100 % percentile ranges span almost the entire density gradient as observed in empirical DNA-SIP datasets (Figure 3 and Pepe-Ranney *et al.* 2016).

How do these fragment BD distributions translate into taxon abundance distributions as observed in actual DNA-SIP datasets? To illustrate, we generated a realistic simulation of amplicon-fragments from all bacterial genomes in our dataset (n = 1147). The simulation included two isopycnic gradients: an unlabeled control and a ^{13}C-labeled treatment community (Figure 2). For the treatment gradient, 10% of the taxa were designated as incorporators with 100% atom excess ^{13}C, while all others had 0% atom excess ^{13}C. For a general comparison, we plotted both the absolute count distributions of DNA fragments in the gradient and the relative abundance distributions that would be observed with 16S rRNA

HTS of 24 gradient fractions (Figure 3). When assessing the absolute abundances of DNA fragments, a BD shift for the distributions of certain taxa can easily be observed by comparing the control and treatment gradients. However, when using relative abundances, as in the case of compositional DNA-SIP datasets, accurately identifying true BD shifts is not straightforward due to compositional effects.

3 Supplemental Tables

Table S1. *Parameters used in the DNA-SIP simulation framework.*

Simulation step	Parameter	Description	Value *
1a) Genomic DNA fragments	F	Fragment size distribution	skewed-normal<br>mu:9000<br>sigma:2500<br>skew:-5
	z	The number of fragments per genome to simulate	10000
1b) DNA fragment length & G+C distributions	h	KDE bandwidth (smoothing parameter)	0.8**
1c) DNA fragment BD probability distributions	β	Beta coefficient based on gradient salt density	1.14E+09
	D	Average particle density in gradient	1.7
	w	Angular velocity of rotor (omega^2)	33172837
	r_min	Radius minimum from axis of rotation (cm)	2.6
	r_max	Radius maximum from axis of rotation (cm)	4.85
	t	Ultracentrifugation run time (seconds)	23760
	T	Ultracentrifugation run temperature (kelvin)	293.15
	G	G coefficient (see Clay et al., 2003)	7.87E-10
	M	Molecular weight per base pair of dry cesium DNA	882
	n	Number of replicates for Monte Carlo error propagation	100000
	h	KDE bandwidth (smoothing parameter)	0.8**
1d) Diffusive boundary layer effects	β	Beta coefficient based on gradient salt density	1.14E+09
	D	Average particle density in gradient (g ml^{-1})	1.7
	w	Angular velocity of rotor (omega^2)	33172781
	tube_diam	Centrifuge tube diameter (cm)	1.3
	tube_height	Centrifuge tube height (cm)	4.8
	r_min	Radius minimum from the axis of rotation (cm)	2.6
	r_max	Radius maximum from the axis of rotation (cm)	4.85
	BD_min	Minimum BD used to determine the DBL (g ml^{-1})	1.59
	BD_max	Maximum BD used to determine the DBL (g ml^{-1})	1.77
	n	Number of replicates for Monte Carlo error propagation	100000
	α	DBL fragments; see Eq. 5	0.001
	γ	Weighting factor; see Eq. 5	0.5**
	h	KDE bandwidth (smoothing parameter)	0.8**
1e) Isotope incorporation	I	Isotope incorporated by taxa	13C
	n	Number of replicates for Monte Carlo error propagation	100000
	h	KDE bandwidth (smoothing parameter)	0.8*

2a) Gradient fractions	d	Distribution used to select fraction BD window sizes	normal
	P	BD window size distribution parameters	mu:0.004<br>sigma:0.0015
	BD_min	Minimum BD of any fraction	1.66
	BD_max	Maximum BD of any fraction	1.78
	frac_min	Minimum fraction size	0.001
2b) Pre-fractionation communities	N	Number of pre-fractionation communities	6
	d	Distribution used for selecting relative abundances	lognormal
	P	Abundance distribution parameters	mu:10<br>sigma:2
	shared_perc	% of taxa shared in each community	100
	perm_perc	% of rank-abundances to permute for each community	0
3a) OTU table	A	Total absolute abundance of taxa in the community	1.00E+09
3b) PCR products	n	Number of PCR cycles	30
	d	Distribution of starting DNA micro-molarities	uniform
	P	DNA concentration distribution parameters	min:0.3<br>max:0.3
	C	Molarity of forward and reverse primers (uM)	1
	r	Amplicon to primer length ratio	10
	f	Theoretical maximum PCR efficiency	1
	k	k parameter used in Suzuki & Giovannoni (1996)	5
3c) Sequence data	d	Distribution used to select number of samples per community	normal
	P	Parameters for the distribution used to select the number of samples per community	mu:20000<br>sigma:5000

* default value, unless otherwise stated in text

** optimized based on empirical data; see Figure S3

Table S2. *Comparison of empirical data* from Leuders, *et al.* 2004 *with simulated results for each taxon with respect to total DNA and 16S rRNA gene amplicons.* BD distribution results were compared for both total DNA and amplicon fragments generated from 16S rRNA genes. The correlation between simulated and empirical results for each DNA type from each taxon was calculated using Pearson's correlation coefficients (*r*). The p-values (*p*) indicate the probability that the correlation is to chance. These results are further illustrated in Figure S1.

**Taxon**	**DNA**	***r***	***p***
M. barkeri MS	Total DNA	0.852	2.16E-04
M. extorquens AM1	Total DNA	0.670	3.26E-03
M. barkeri MS	16S rRNA genes	0.888	8.87E-07
M. extorquens AM1	16S rRNA genes	0.970	3.27E-11

4 Supplementary Figures

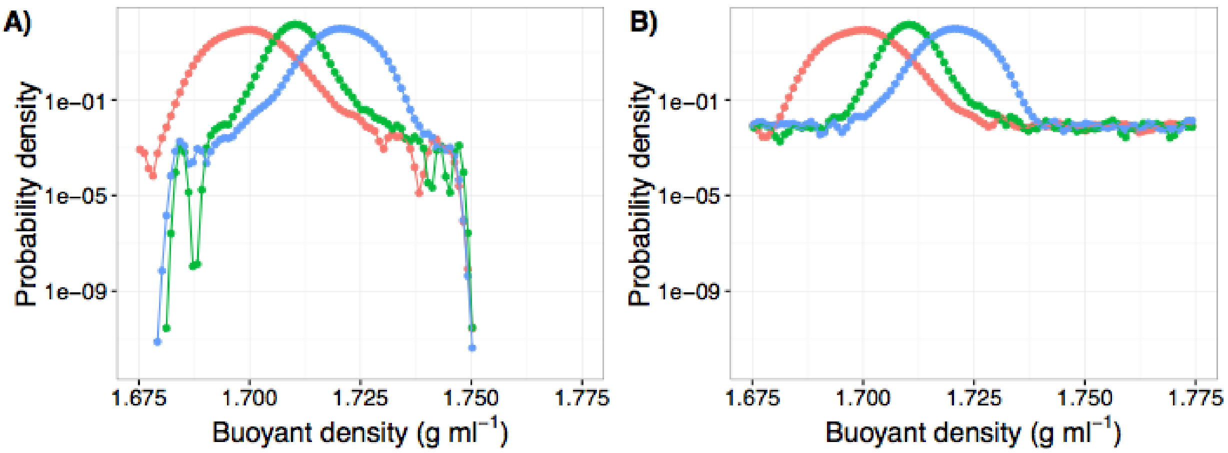

Figure S1. *Fragment distributions that are strictly Gaussian (A) predict very low probability of detecting DNA at either end of the CsCl density gradient, while these probabilities are increased dramatically by including diffusive boundary layer effects (B).* Contrasting simulations were run without (A) and with (B) including diffusive boundary layer (DBL) effects on amplicon-fragment BD distributions. This simulation includes DNA fragments from *Clostridium ljungdahlii* DSM 13528 (red), *Escherichia coli* 1303 (green), and *Streptomyces pratensis* ATCC 33331 (blue). Fragment sizes were drawn from a distribution as described in Table S1. Note that excluding DBL effects results in extremely low probability densities at either end of the gradient, while inclusion of DBL effects increases the likelihood with which DNA fragments will be observed throughout the gradient. See also Figures S16 and S17 which show additional characteristics of the effects of DNA fragment size and distribution parameters for these three genomes, respectively.

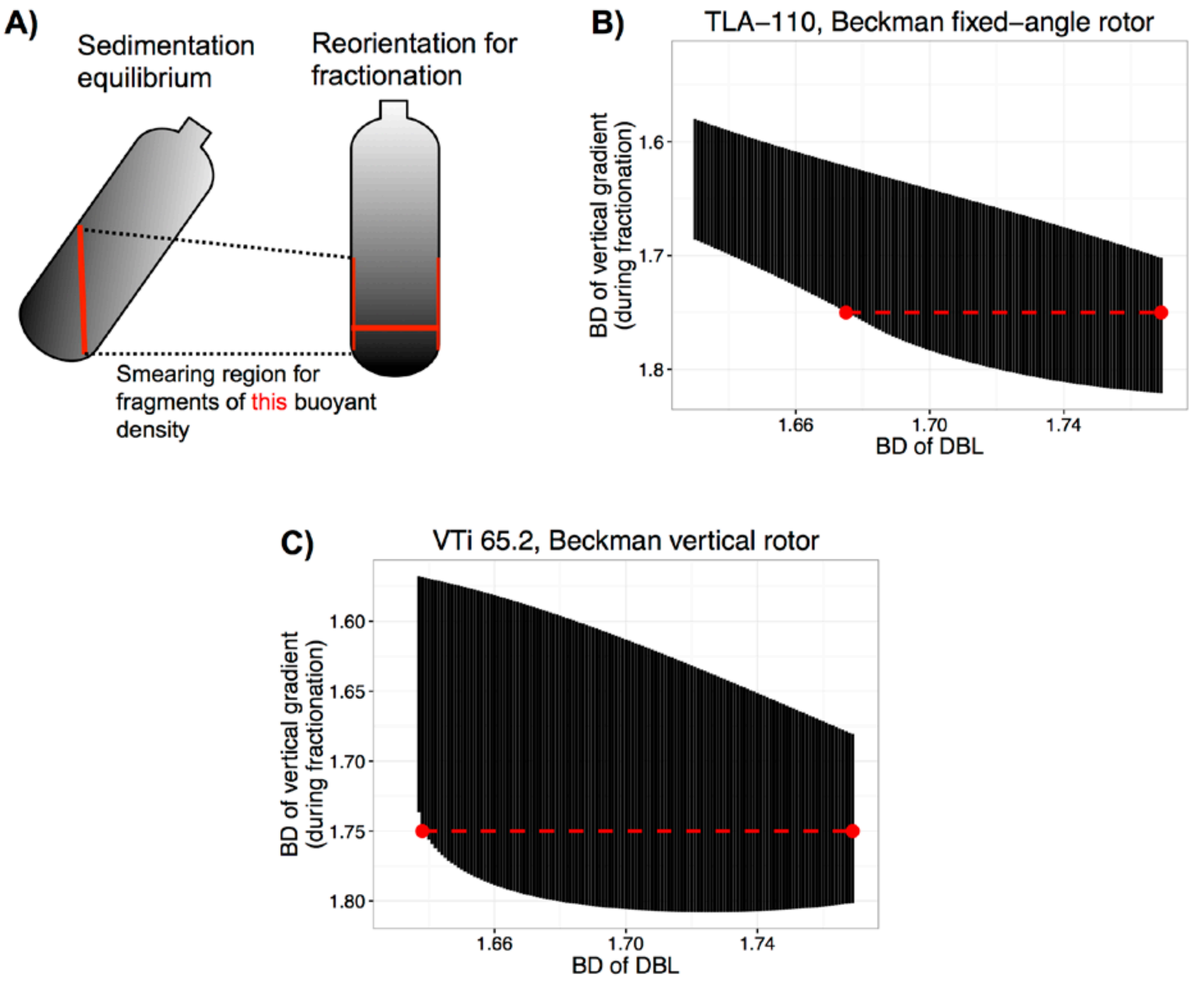

Figure S2. *An example of diffusive boundary layer (DBL) effects for an angled or vertical rotor.* The isopycnic gradient forms perpendicular to the axis of rotation during ultracentrifugation but reorients to the axis of gravitational pull during centrifuge deceleration, which is then followed by gradient fractionation. During sedimentation equilibrium (A), the BD of DNA in the boundary layer will equal the BD of DNA in the lumen of the tube. However, following gradient reorientation, the DBL will entrain DNA in the boundary layer causing the boundary layer DNA to differ in BD from the DNA in the lumen. The solid red lines indicate the position of DNA molecules that have identical BD when both at sedimentation equilibrium and after reorientation (A). The red dashed line in (B) and (C) illustrates the BD range of boundary layer DNA that would be present in a gradient fraction whose lumen has a BD of the 1.75 g ml^{-1} following gradient reorientation. It is notable that the effect of boundary layer DNA is far greater in vertical (C) than in fixed angle rotors (B).

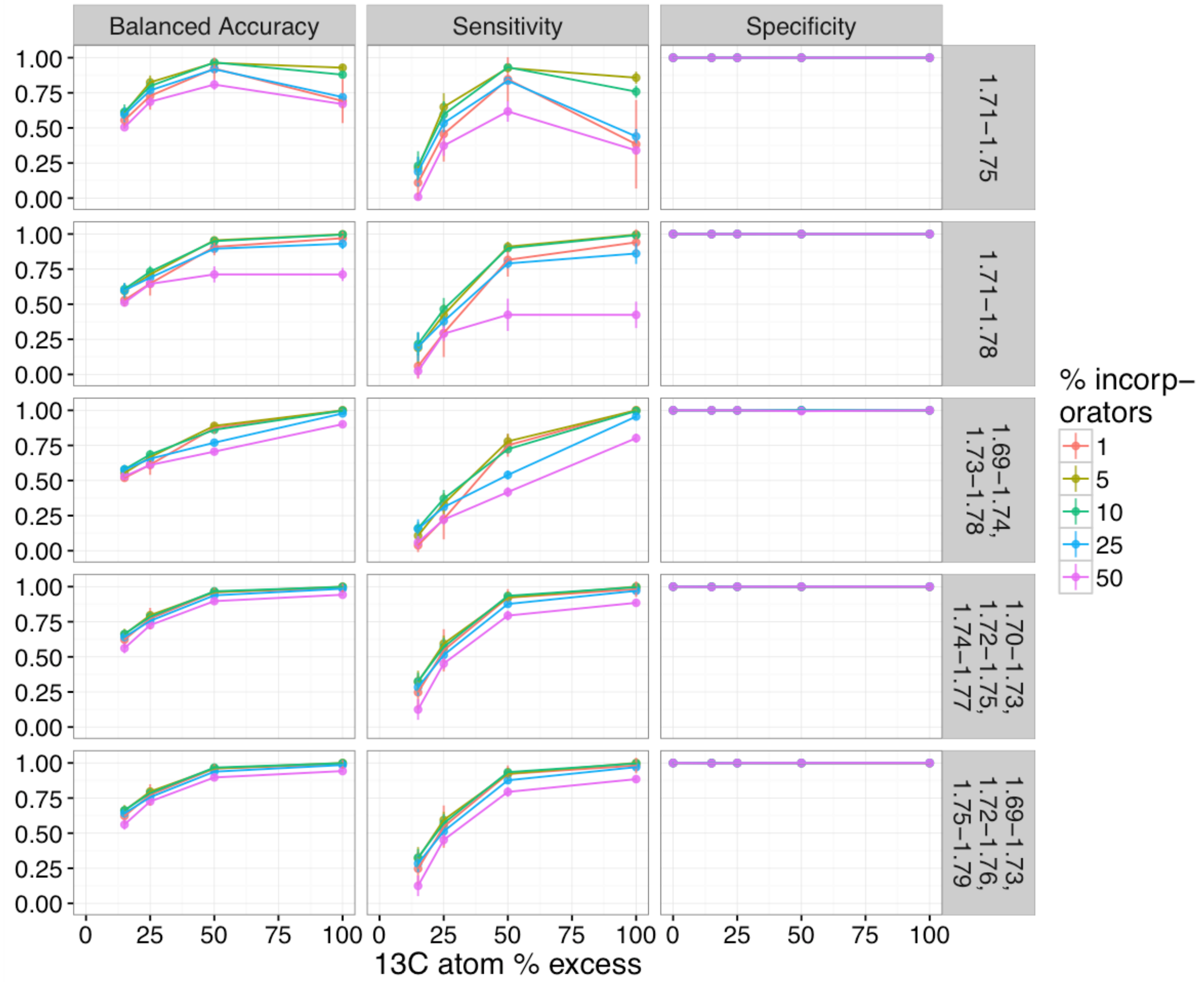

Figure S3. *Utilizing multiple "heavy" BD windows for HR-SIP can improve sensitivity while having little effect on specificity.* HR-SIP as used in Pepe-Ranney *et al.,* (2016) compares differential abundance between isotopically enriched treatment samples and unenriched control samples across a single BD window (1.71 – 1.75 g/ml). The use of multiple windows (MW-HR-SIP; bottom 3 rows) provides greater sensitivity than the use of a single window (HR-SIP; top 2 rows) without compromising specificity. Points represent the mean values among 10 simulation replicates, and the error bars are standard deviations. The plots are faceted on the y-axis by the BD window(s) used in the analysis.

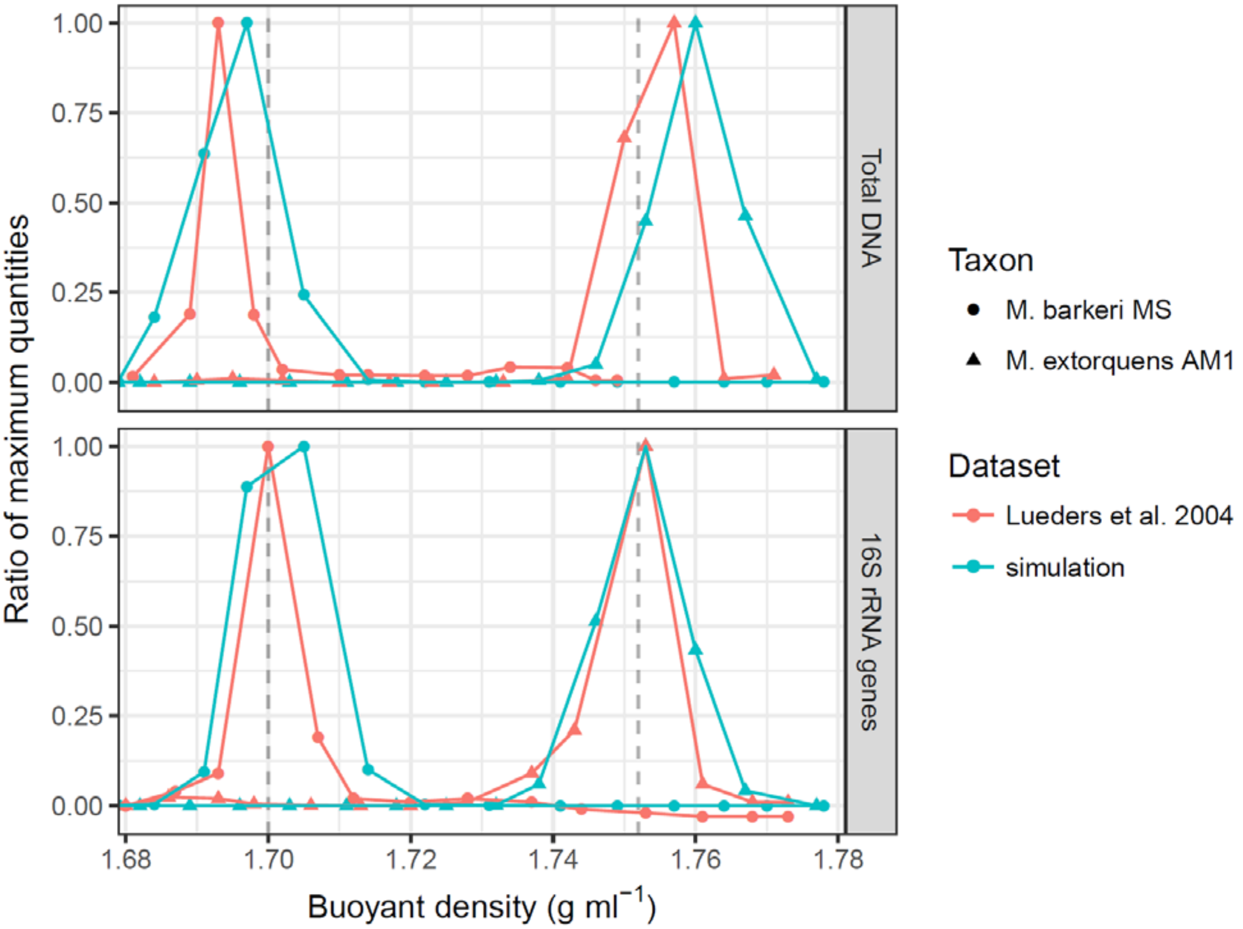

Figure S4. *SIPSim closely replicates empirical data in Lueders et al., (2004)*. A simulated recreation of empirical DNA-SIP data from Figure 1 of Leuders et al., (2004). The "Lueders et al. 2004" data was reproduced from Lueders *et al.,* (2004). "Total DNA" shows the relative abundances of total genomic fragments as was measured in Lueders et al., (2004) by a PicoGreen dsDNA quantification assay. "16S rRNA genes" shows the relative abundances of 16S rRNA amplicon-fragments as measured in Leuders et al., (2004) by qPCR. See Table S2 for statistical comparisons between datasets. Dashed lines are added to aid visual comparison between upper and lower panels.

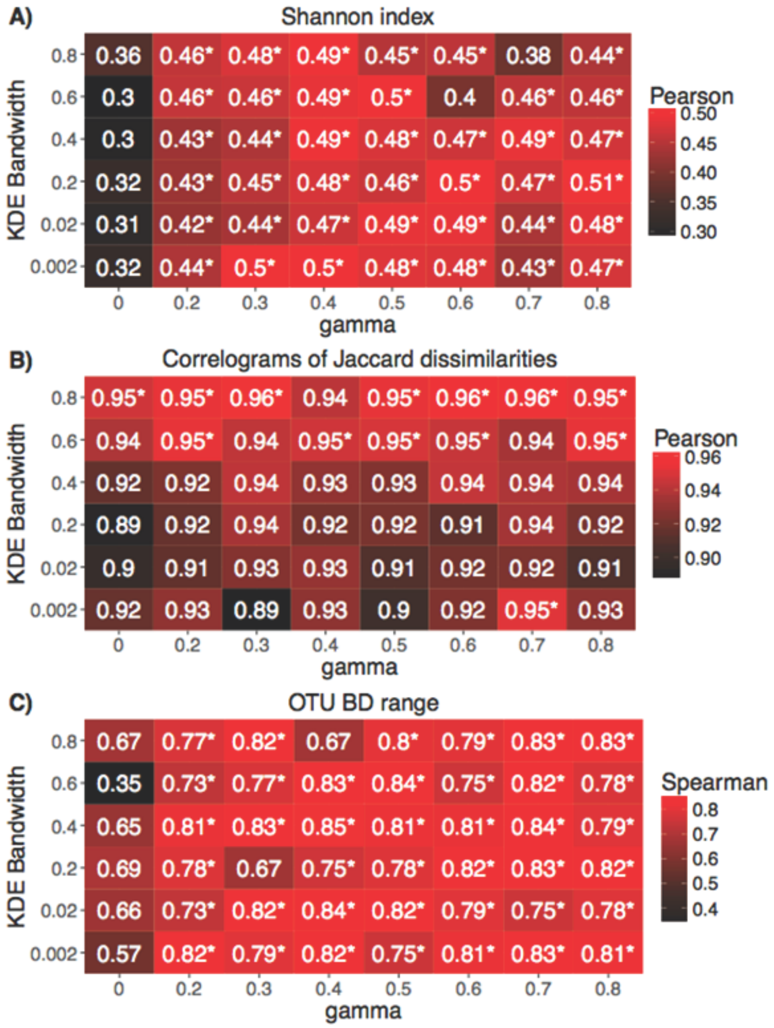

Figure S5. *Comparison of empirical results (as shown in Figure 3) with simulated results indicates that the SIPSim model can accurately reproduce the BD characteristics of complex mixtures of DNA fragments equilibrated in CsCl density gradients.* Shannon index values were compared (using the Pearson correlation coefficient, as described in Figure S14) between corresponding gradient fractions of empirical and simulated CsCl density gradients (A). In addition, Jaccard dissimilarity values were compared (using Pearson correlation coefficients of correlograms, as described in Figure S15) between real and simulated gradient fractions (B). Finally, BD range characteristics were compared (using Spearman correlation coefficients measuring similarity in the relationship between OTU BD range and pre-fractionation relative abundance, as depicted in Figure 3) between corresponding gradient fractions of real and simulated gradient fractions (C). "KDE bandwidth" is the bandwidth (smoothing parameter) used for kernel density estimation during the DNA-SIP simulations, and "gamma" (γ) is a parameter that determines the effects of the diffusive boundary layer (see Eq. 5). The heatmap values for all panels correspond with values reported as numerals. Asterisks indicate values for which the correlation coefficient is significantly greater between simulated and empirical data than between empirical replicates.

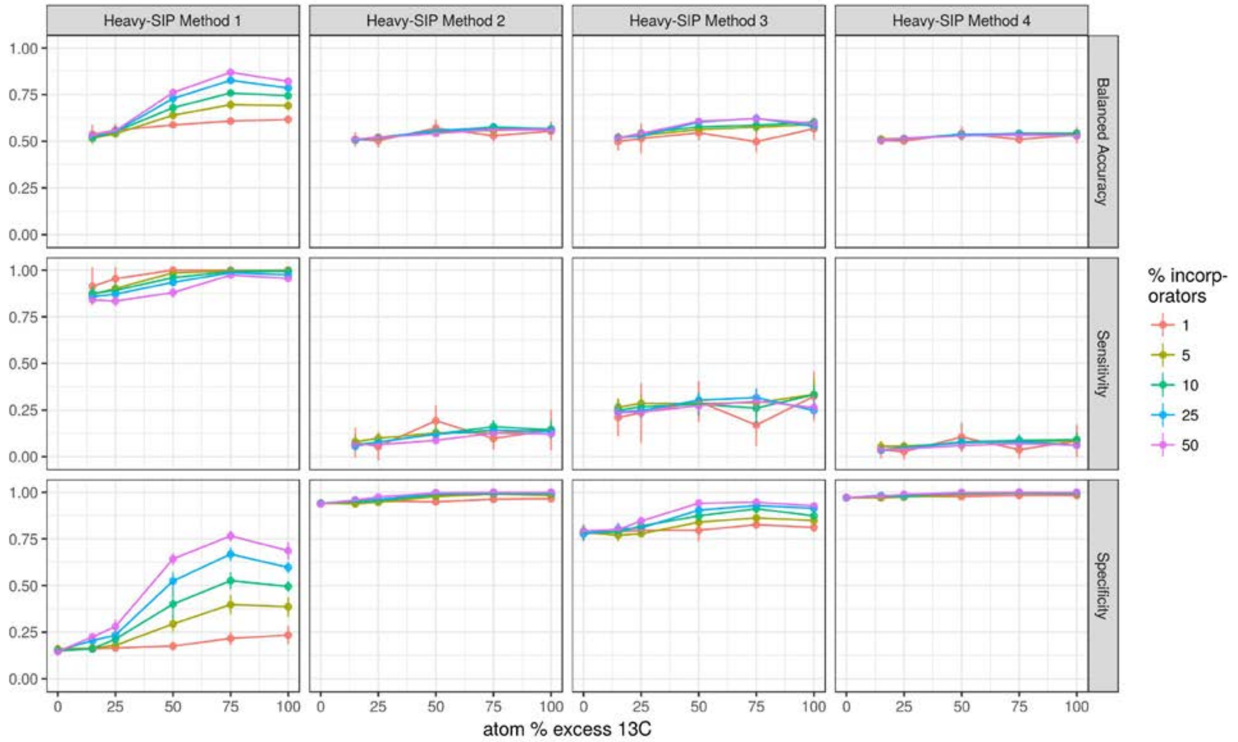

Figure S6. *Four different Heavy-SIP approaches were shown to have fairly low balanced accuracy in identifying labeled OTUs relative to competing methods (as shown in Figure 4).* Method 1 (as shown depicting Heavy-SIP in Figure 4) identifies as labeled any taxa present in "heavy" fractions of the labeled treatment gradients. Method 2 identifies as labeled any taxa present in the "heavy" fractions of the labeled treatment and absent from the "heavy" fractions of the control gradient. Method 3 identifies as labeled any taxa present in the "heavy" fractions of the labeled treatment and absent in the "light" fractions of the labeled treatment. Method 4 identifies as labeled any taxa present in the "heavy" fractions of the labeled treatment and absent from both the "heavy" fractions of the control and the "light" fractions of the labeled treatment. Points and bars represent means and standard deviations, respectively (n = 10 simulations). Specificity indicates the fraction of true negatives that are identified correctly. Sensitivity indicates the fraction of labeled taxa (true positives) identified correctly. Balanced accuracy is the product of specificity and sensitivity. The x-axis indicates the amount of ^{13}C isotope present in taxa that are labeled, and different colors are used to indicate the percentage of taxa that have incorporated ^{13}C
as indicated by the legend.

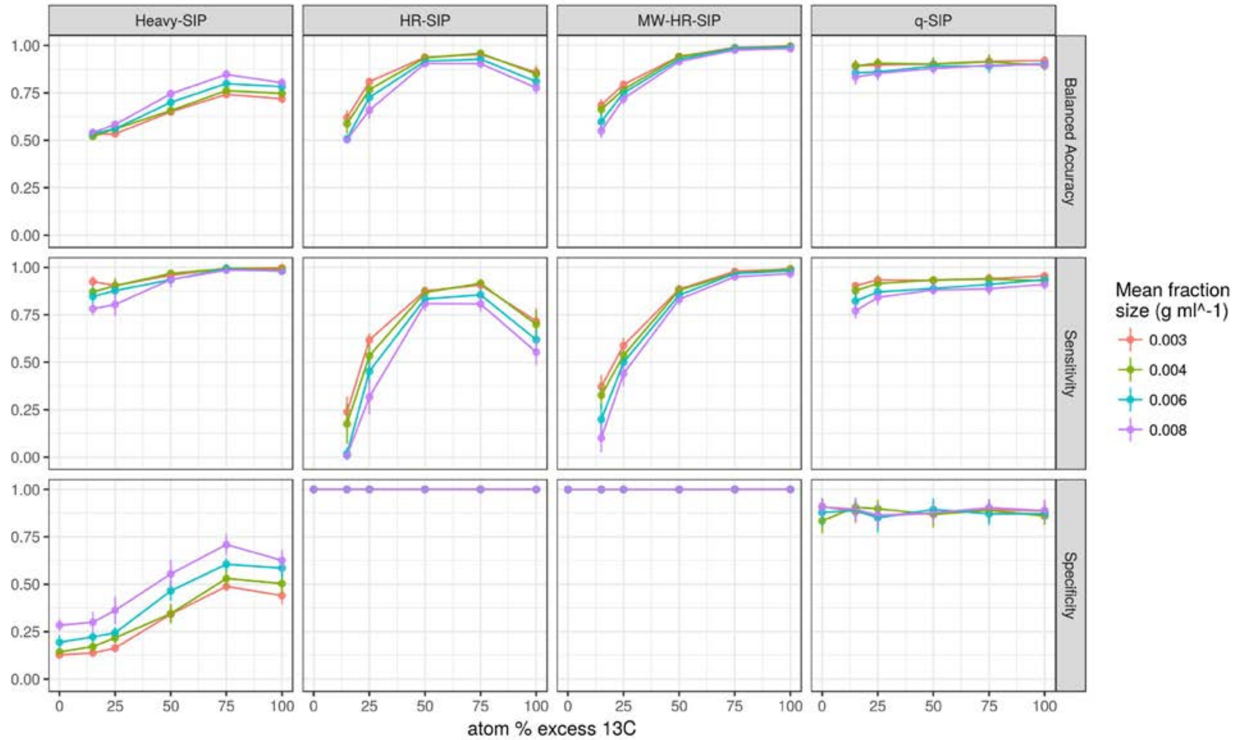

Figure S7. *DNA-SIP methods vary in accuracy depending on the size of gradient fractions analyzed.* Specificity indicates the fraction of true negatives that are identified correctly. Sensitivity indicates the fraction of labeled taxa (true positives) identified correctly. Balanced accuracy is the product of specificity and sensitivity. The *x*-axis indicates the amount of ^{13}C isotope present in taxa that are labeled, and different colors are used to indicate the density range captured by each gradient fraction as indicated by the legend. For reference, a gradient that spanned a buoyant density range of 1.66 to 1.75 g ml^{-1} would have approximately 30, 22, 15, and 11 fractions of mean size 0.003, 0.004, 0.006, and 0.008 g ml^{-1}, respectively. Points and bars represent means and standard deviations, respectively (n = 10 simulations).

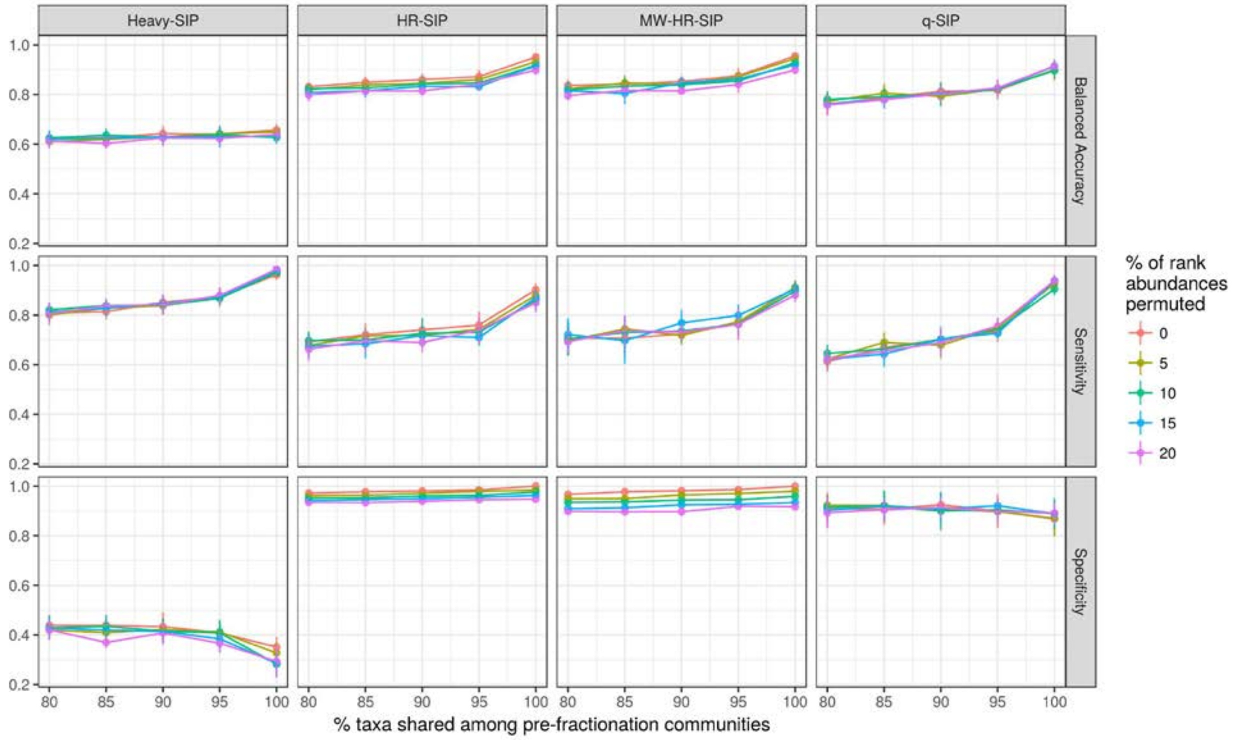

Figure S8. *DNA-SIP methods are differentially affected by increasing the beta diversity between unlabeled controls and isotopically labeled treatments*. Beta diversity was varied among the 6 pre-fractionated communities in each simulation (3 replicate ^{12}C and ^{13}C gradients). Points represent mean values among 10 simulation replicates, and the error bars are ± the standard deviation. For all simulations, 10% of the community were incorporators (50% atom excess ^{13}C).

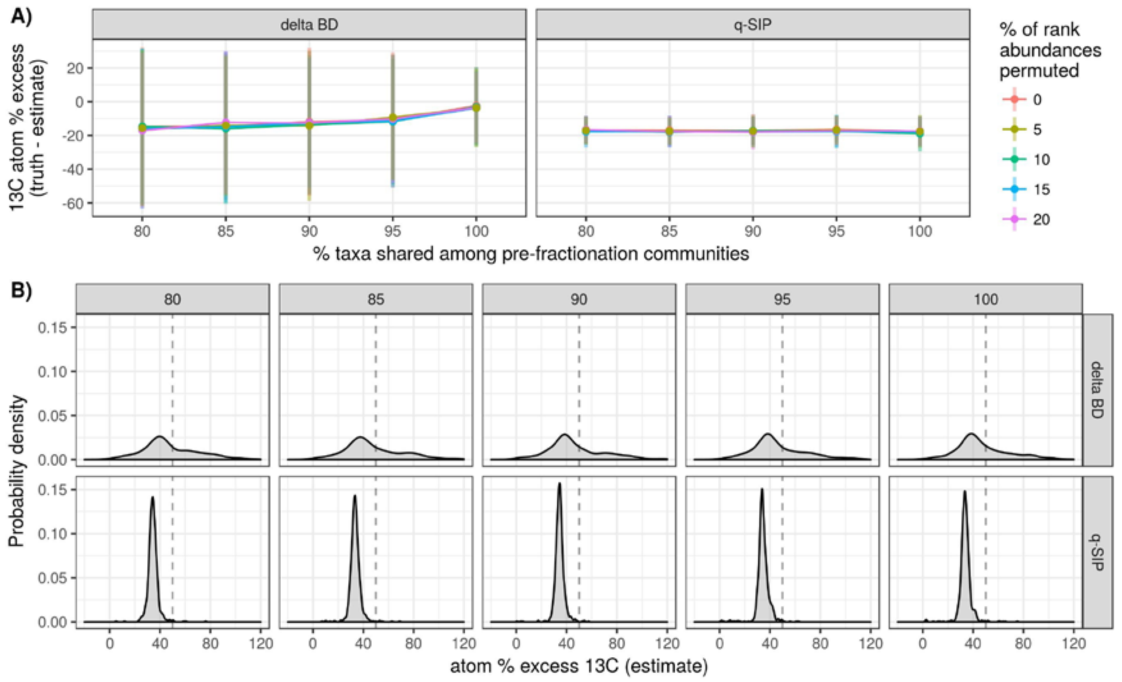

Figure S9. *ΔBD but not q-SIP estimates of % atom enrichment of DNA are substantially affected by beta diversity among replicate samples.* For the plots in (A), points represent the mean values among 10 simulation replicates, and bars indicate standard deviations. The kernel density plots of estimated atom % excess ^{13}C in (B) highlight the variability between methods as shown in (A). The x-axis facet labels are the percent taxa shared as in (A), and only distributions for simulations of 10% taxa ranks permuted are shown in (B).

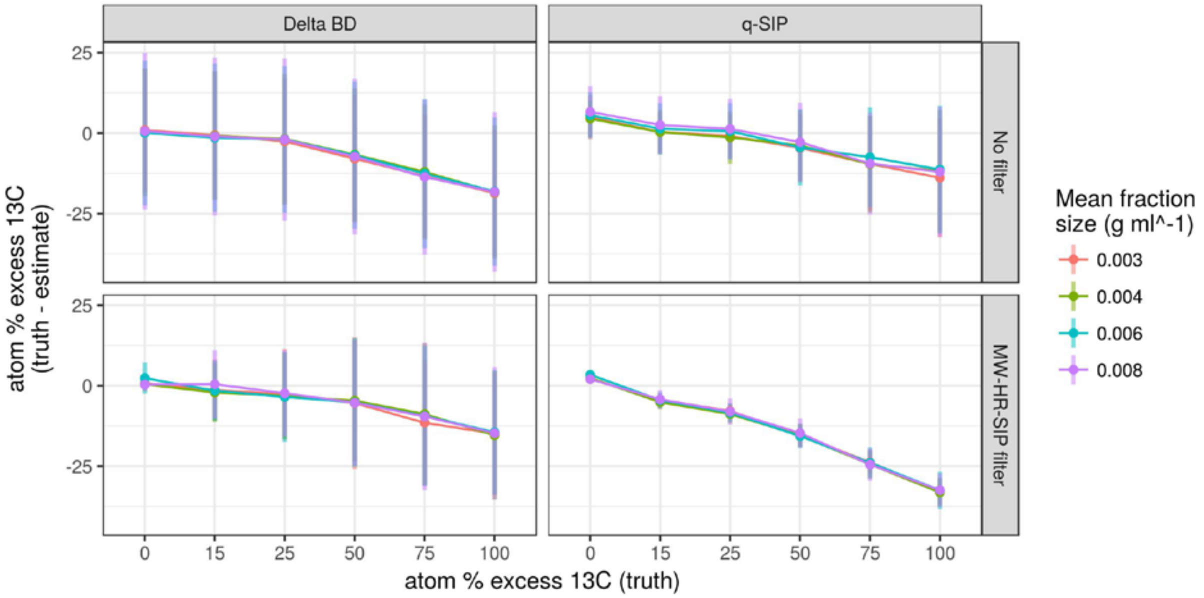

Figure S10. *The size of gradient fractions has little effect on estimates of atom % excess ^{13}C.* The difference between actual (truth) and estimated atom % excess ^{13}C is indicated on the *x*-axis while the level of atom % excess ^{13}C labeling is varied along the *y*-axis. Results are shown both for all taxa (no filter, top panels), and only taxa first identified as labeled by MW-HR-SIP (bottom panels). This inclusion of unlabeled taxa in the top panels tends to increase variance. Different colors are used to indicate the use of different gradient fraction sizes as indicated in the legend. Values indicate the mean and standard deviation (n = 10 simulations).

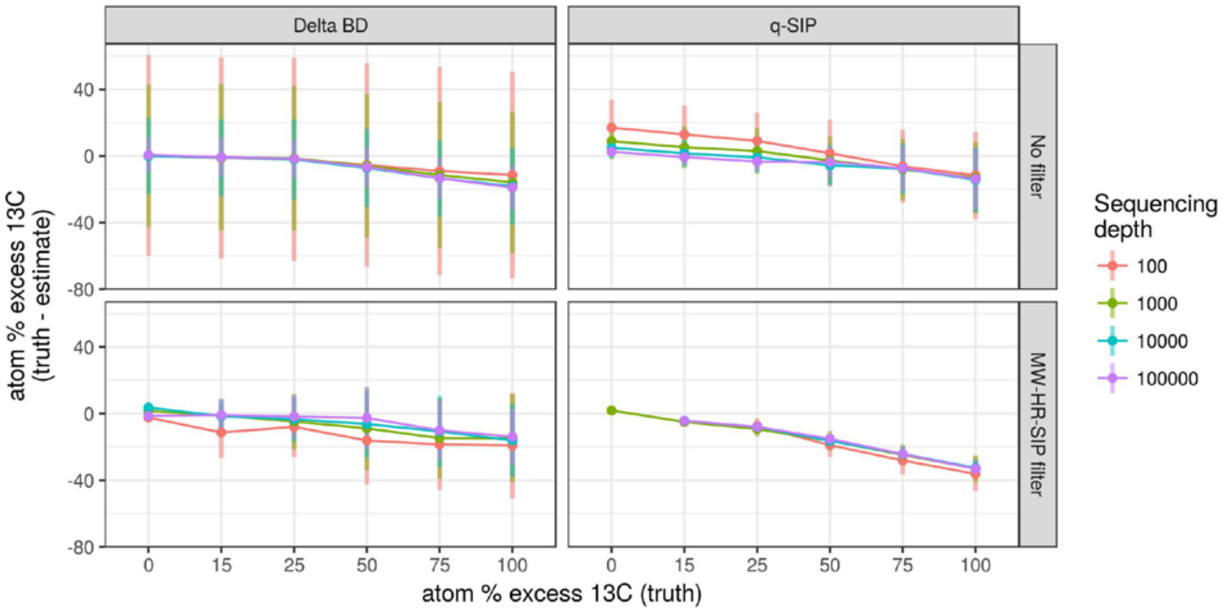

Figure S11. *Sequencing depth has little effect on estimates of atom % excess ^{13}C.* The difference between actual (truth) and estimated atom % excess ^{13}C is indicated on the *x*-axis while the level of atom % excess ^{13}C labeling is varied along the *y*-axis. Results are shown both for all taxa (no filter, top panels), and only taxa first identified as labeled by MW-HR-SIP (bottom panels). This inclusion of unlabeled taxa in the top panels tends to increase variance. Different colors are used to indicate the use of different sequencing depth in the legend. Values indicate the mean and standard deviation (*n* = 10 simulations).

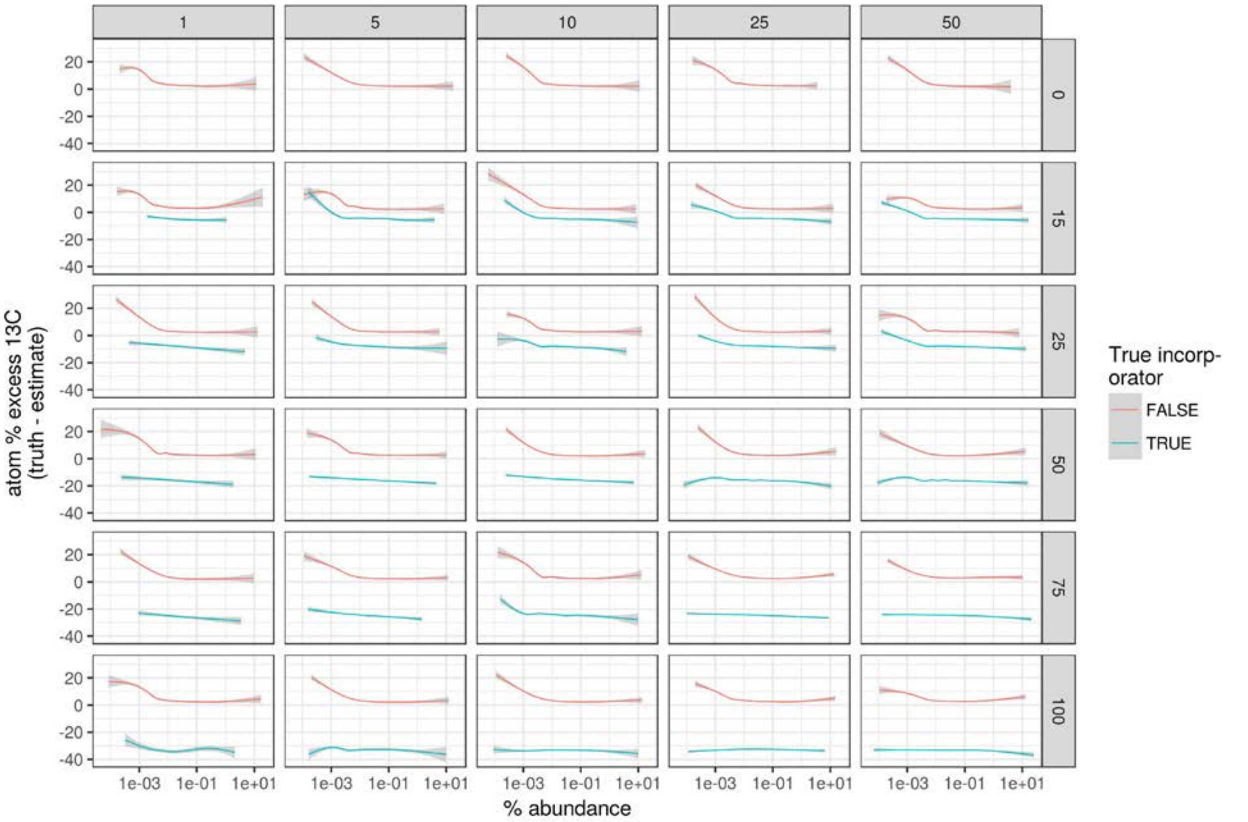

Figure S12. *The degree to which qSIP underestimates atom % excess ^{13}C tends to increase with the true value of atom % excess ^{13}C and is little affected by the relative abundance or the number of taxa that incorporate isotope.* Each plot shows the difference between actual (truth) and estimated atom % excess ^{13}C in relation to OTU relative abundance. The total number of labeled OTUs is varied by column and the level of isotopic enrichment is varied across rows. Blue lines show values for labeled taxa while red lines indicate results for unlabeled taxa. Note, that the results are unaffected by the rate of true and false positives since the all OTUs are being assessed and not just those found to be incorporators by qSIP. Lines are LOESS curves fit to values for all simulations, and the grey regions represent 99% confidence intervals.

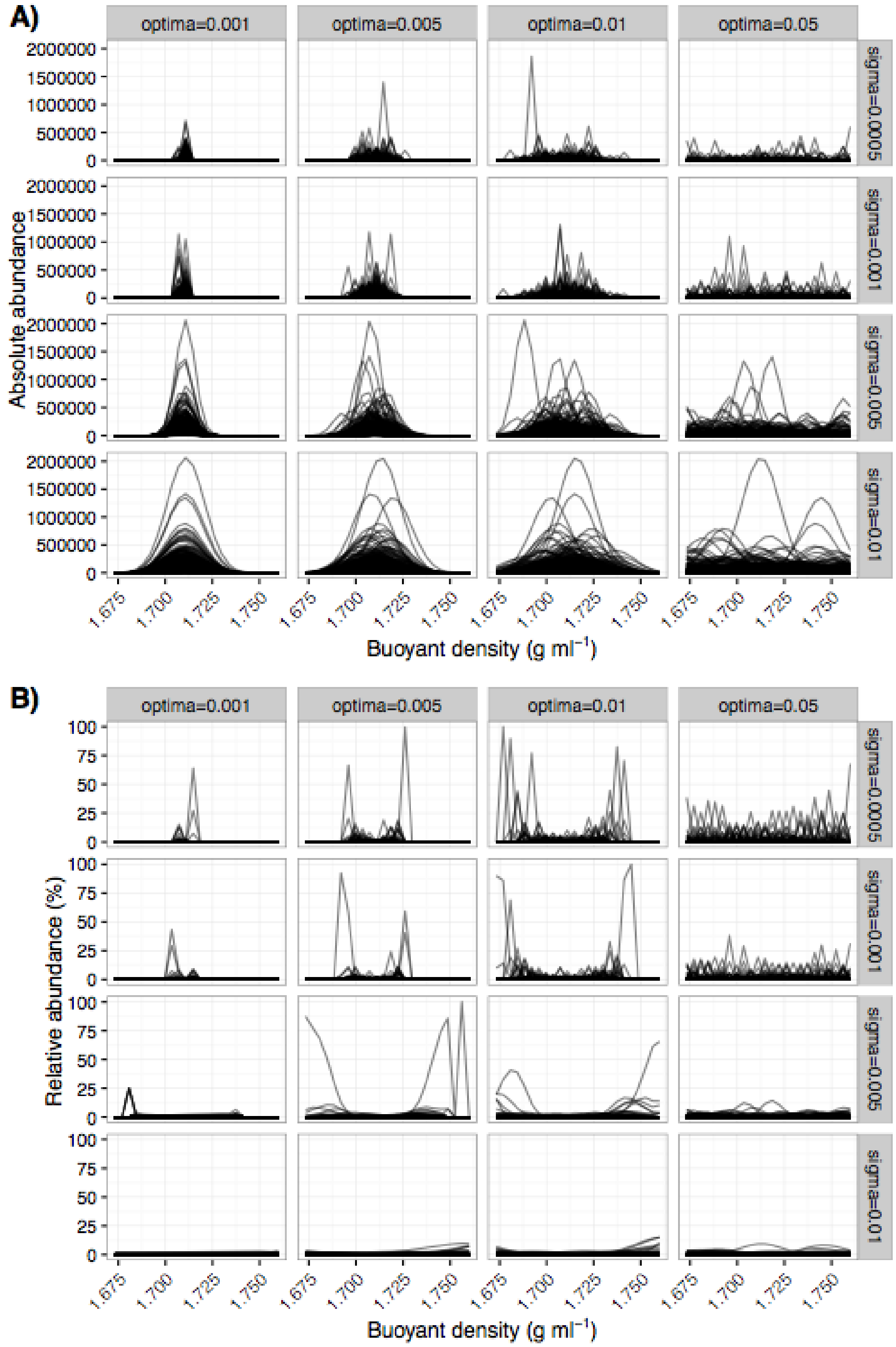

Figure S13. *Gradient community simulations with Gaussian fragment BD distributions.* Fragment BD distributions (*n*=1200) were simulated for unlabeled DNA by varying 2 parameters: sigma and optima, which are the standard deviation of the distributions (distribution "width") and the location of the distribution optimum, respectively. The distributions are shown as absolute abundances (A) or transformed into compositional data (*i.e.* relative abundances) in (B).

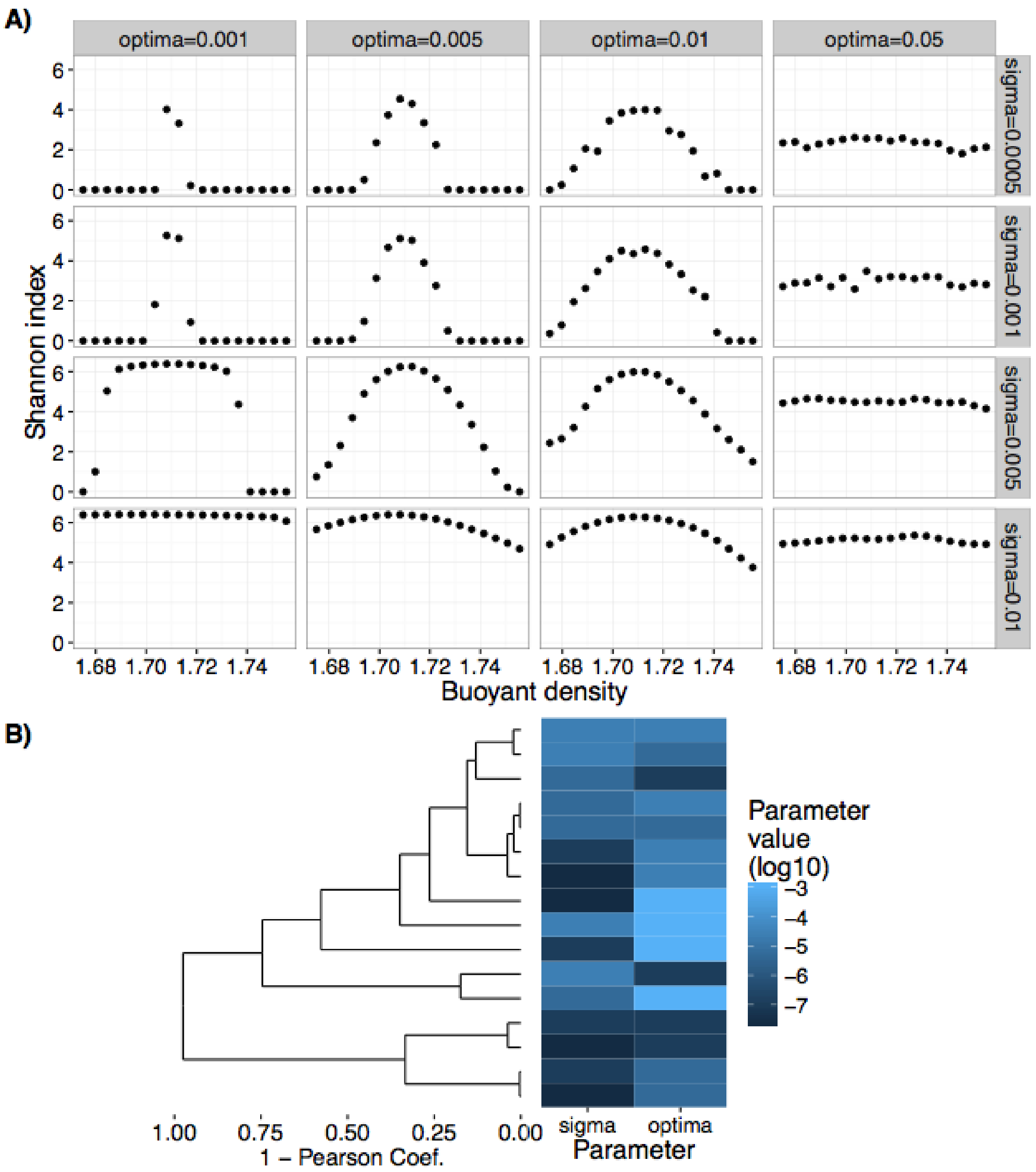

Figure S14. *The Shannon index can be used to distinguish differences in fragment BD distributions transformed into compositional datasets (i.e. DNA-SIP datasets).* Each plot in (A) represents Shannon index values calculated from the simulated communities shown in Figure S13. The distributions of Shannon index values (Shannon index ~ BD) for each simulation (each sigma:optima) were compared in pairwise fashion by calculating Pearson correlation coefficients to determine whether simulations with similar fragment BD distributions produce similar Shannon index value distributions. These pairwise Pearson correlation coefficients are depicted by the dendrogram in (B).

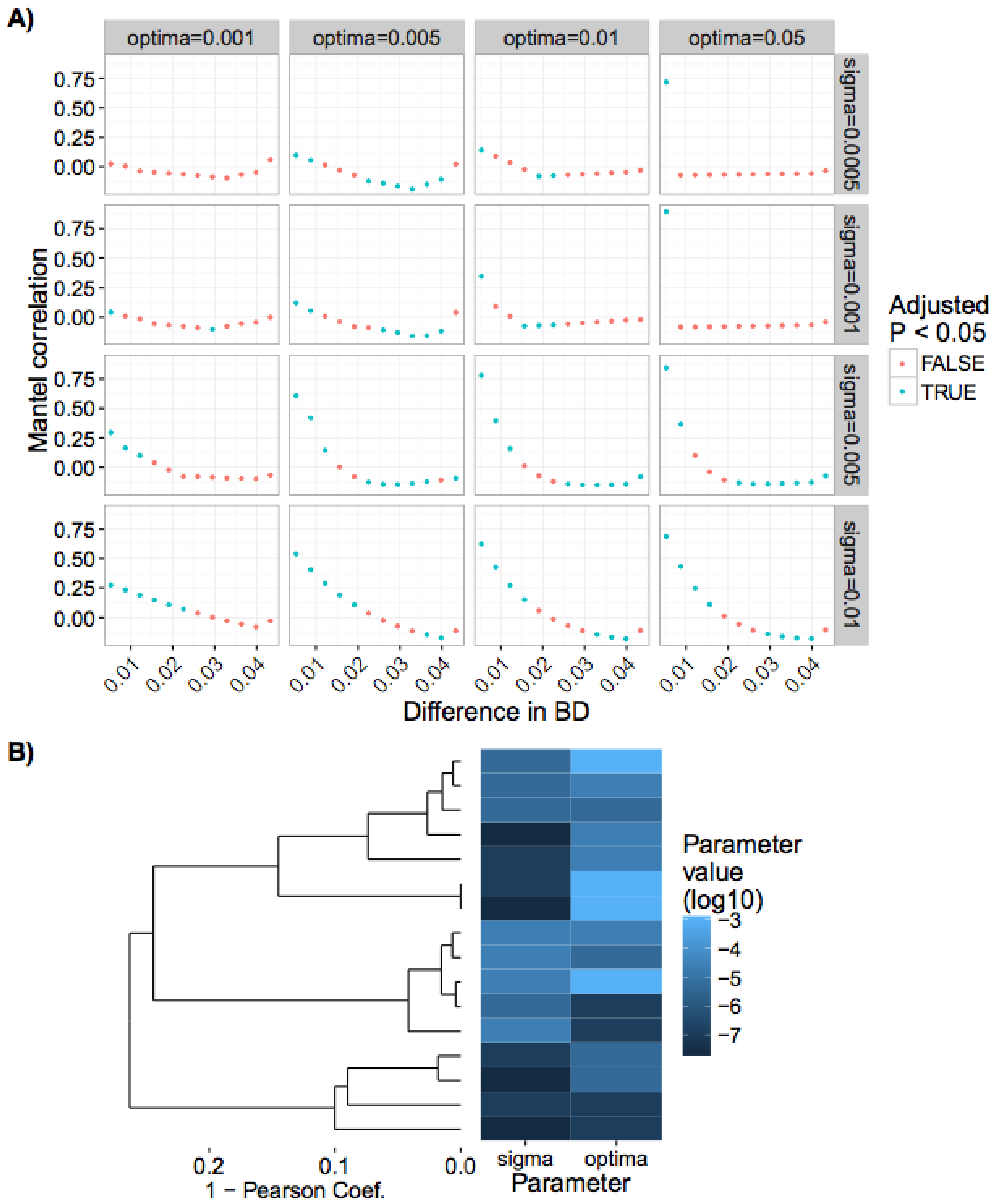

Figure S15. *Correlograms which assess autocorrelation of DNA similarity across gradient fractions can be used to distinguish differences in fragment BD distributions transformed into compositional data (i.e. DNA-SIP datasets).* Each plot in (A) is a correlogram of Jaccard index values calculated from the simulated communities shown in Figure S13. The correlograms depict the amount of autocorrelation (measured by Mantel correlation) in taxonomic similarity as a function of the BD difference between fractions. The distribution of Mantel correlation values (Mantel ~ Difference in BD) for each community (each sigma:optima) were compared in pairwise fashion by calculating Pearson correlation coefficients in order to determine whether communities with similar fragment BD distributions produce similar correlograms. These pairwise Pearson correlation coefficients are depicted by the dendogram in (B).

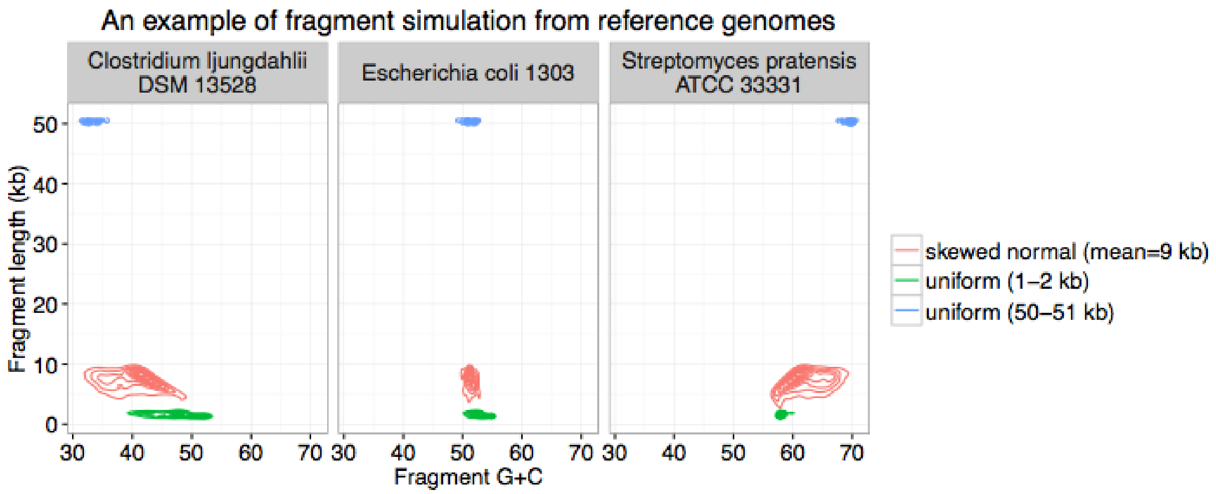

Figure S16. *A change in DNA fragment-length distribution impacts the G+C content of genome fragments that contain SSU rRNA genes (amplicon-fragments)*. Different DNA extraction methods cause different amounts of DNA shearing, which results in different DNA fragment length distributions. The figure shows the 2-dimensional Gaussian kernel density estimations of fragment length and G + C content for sheared genomic DNA from the genomes *Clostridium ljungdahlii* DSM 13528, *Escherichia coli 1303*, and *Streptomyces pratensis* ATCC 33331, which have genome G + C contents of 31.1, 50.7, and 71.1, respectively. The "skewed normal (mean = 9 kb)" distribution represents a typical DNA length distribution from bead-beating methods. The "uniform (1-2 kb)" and "uniform (50-51 kb)" are representative of low and high molecular weight DNA, respectively.

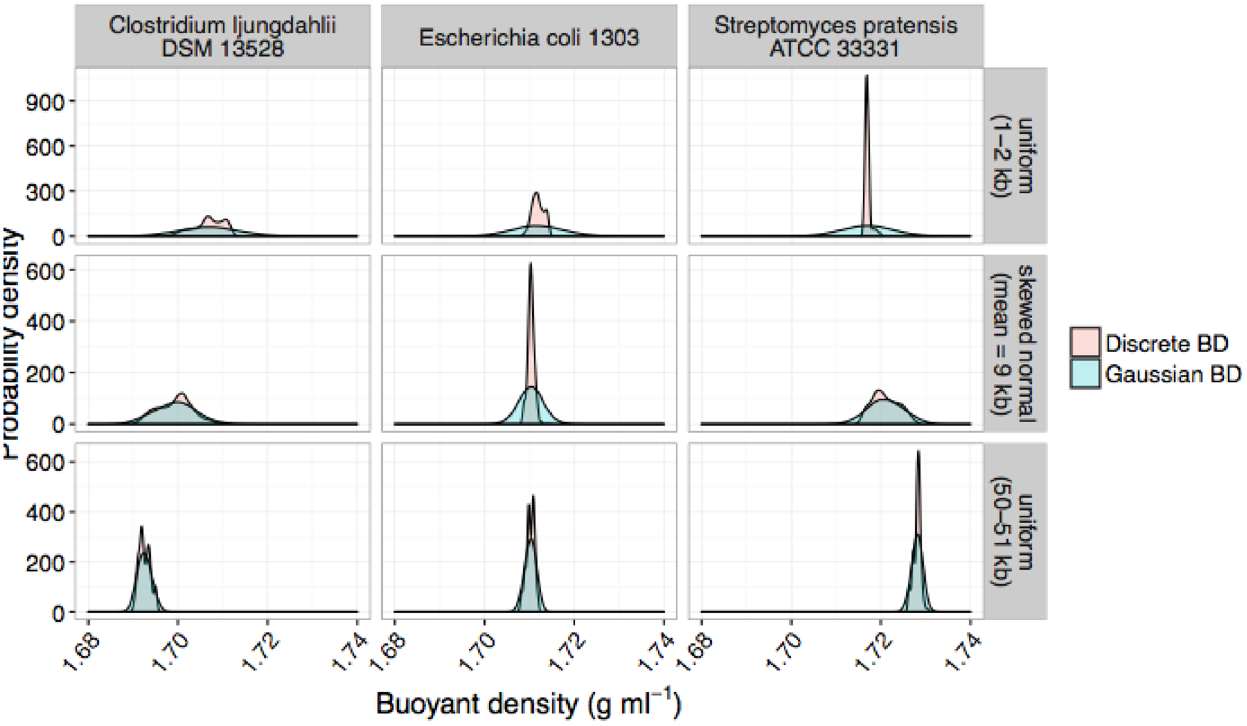

Figure S17. *An example of how amplicon-fragment BD distributions can vary depending on whether fragment BD values are modeled as in Eq. 1 ("Discrete BD") or Eq. 3 ("Gaussian BD")*. Plots were faceted by the fragment length distribution (see Figure S16). Note that accounting for the effects of both DNA shearing and the Gaussian nature of diffusion causes DNA fragments to be found across a much wider range of buoyant densities than would be predicted based on the assumption of uniform high MW DNA fragments with discrete BDs calculated solely from G + C content.

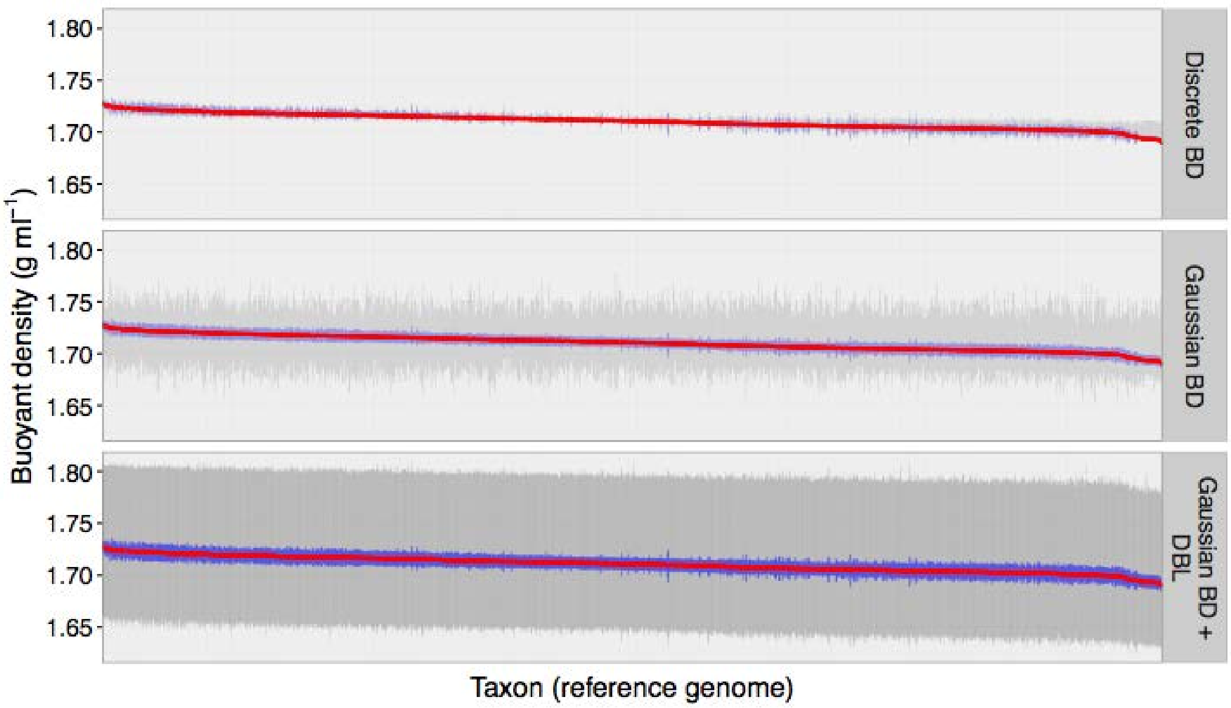

Figure S18. *Amplicon-fragment BD distributions are greatly affected by diffusion and diffusive boundary layer effects.* The plots show amplicon-fragment BD distributions for DNA fragments simulated from the bacterial genomes dataset (*n* = 1147). The median of each BD distribution is signified by a red point, while the 5-95 and the 0-100 percentile ranges are represented as blue and grey lines, respectively. The "Discrete BD" simulation models fragment BD according to Eq. 1. The "Gaussian BD" models DNA fragments as collections of Gaussian distributions with a mean determined from Eq. 1 and standard deviation determined from Eq. 3. The "Gaussian BD + DBL" simulation adds diffusive boundary layer effects to the "Gaussian BD" model. The final model most closely matches empirical results (Figure 3).

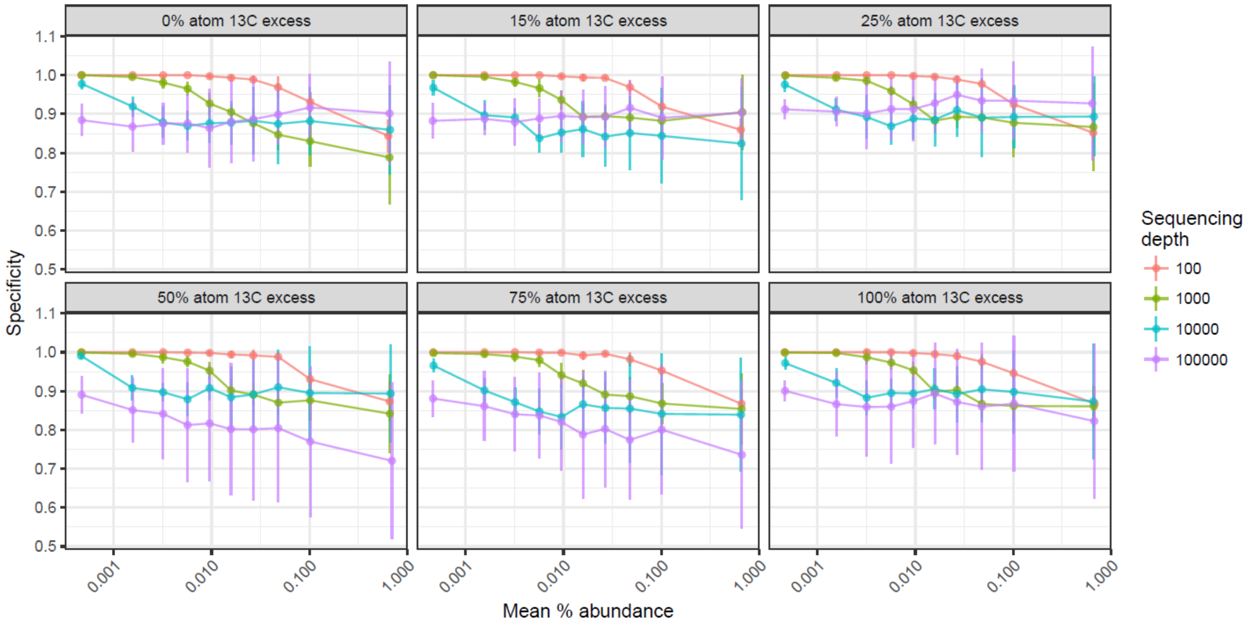

Figure S19. *The specificity with which qSIP detects OTUs is not strongly effected by OTU relative abundance across a range of isotope incorporation*. This result suggests that the use of relative abundance cutoffs to exclude low abundance OTUs will have little impact on the rate of false positives. Furthermore we observe that specificity declines with increasing sequencing effort, and this result is expected if the false positive rate for qSIP is impacted by the number of hypotheses being tested. Each panel provides results from simulations conducted at different levels of atom % ^{13}C enrichment as indicated (0, 15, 25, 50, 75, and 100 atom % ^{13}C), incorporators were identified using qSIP, and sensitivity was assessed by binning OTUs into 10 different abundance classes. The *x*-axis indicates the mean relative abundance of the taxa being evaluated, and different colors are used to indicate the average number of sequences determined per gradient fraction as described by the legend. Points and bars represent means and standard deviations, respectively (n = 10 simulations).

5 Supplementary References

Jones, Eric, Travis Oliphant, Pearu Peterson, and Others (2016) SciPy: Open source scientific tools for Python. http://www.scipy.org/.

McKerns, M. M., L. Strand, T. Sullivan, A. Fang, and Mag Aivazis (2011) Building a framework for predictive science. http://arxiv.org/pdf/1202.1056.

McMurdie, Paul J., and Susan Holmes. (2013) Phyloseq: an R package for reproducible interactive analysis and graphics of microbiome census data. *PLoS ONE*. http://dx.plos.org/10.1371/journal.pone.0061217.

Simpson, Gavin L. (2015) Coenocliner: a coenocline simulation package for R. Regina, Canada.

Wickham, Hadley (2009) ggplot2: Elegant Graphics for Data Analysis. New York: Springer.

Wickham, Hadley (2016) Tidyr: easily tidy data with `spread()` and `gather()` functions. https://CRAN.R-project.org/package=tidyr.

Wickham, Hadley, and Romain Francois (2016) Dplyr: A grammar of data manipulation. https://CRAN.R-project.org/package=dplyr.
